# The impact of swidden decline on livelihoods and ecosystem services in Southeast Asia: A review of the evidence from 1990 to 2015

**DOI:** 10.1007/s13280-016-0836-z

**Published:** 2016-11-16

**Authors:** Wolfram H. Dressler, David Wilson, Jessica Clendenning, Rob Cramb, Rodney Keenan, Sango Mahanty, Thilde Bech Bruun, Ole Mertz, Rodel D. Lasco

**Affiliations:** 1 School of Geography, University of Melbourne, 221 Bouverie, Parkville, VIC 3070 Australia; 2 World Agroforestry Centre (ICRAF), International Rice Research Institute, UPLB, College, Philippines Office, 2nd Floor, Khush Hall Building, PO Box 35024, 4031 Los Baños, Laguna Philippines; 3 Itad, Unit 12 English Business Park, Hove, UK; 4 Center for International Forestry Research (CIFOR) Headquarters, Jalan CIFOR, Situ Gede, Sindang Barang, Bogor, Indonesia; 5 School of Agriculture and Food Sciences, University of Queensland, Hartley Teakle Building (83) Room S511, St Lucia Campus, Brisbane, St Lucia, QLD 4072 Australia; 6 Crawford School of Public Policy, College of Asia and the Pacific, Australian National University, Canberra, Australia; 7 Department of Geosciences and Natural Resource Management, University of Copenhagen, Øster Voldgade 10, 1350 Copenhagen K, Denmark; 8 School of Ecosystem and Forest Sciences, University of Melbourne, Baldwin Spencer Building, Parkville, VIC 3010 Australia

**Keywords:** Alternative land uses, Ecosystem services, Livelihood security, Shifting cultivation, Southeast Asia

## Abstract

Global economic change and policy interventions are driving transitions from long-fallow swidden (LFS) systems to alternative land uses in Southeast Asia’s uplands. This study presents a systematic review of how these transitions impact upon livelihoods and ecosystem services in the region. Over 17 000 studies published between 1950 and 2015 were narrowed, based on relevance and quality, to 93 studies for further analysis. Our analysis of land-use transitions from swidden to intensified cropping systems showed several outcomes: more households had increased overall income, but these benefits came at significant cost such as reductions of customary practice, socio-economic wellbeing, livelihood options, and staple yields. Examining the effects of transitions on soil properties revealed negative impacts on soil organic carbon, cation-exchange capacity, and aboveground carbon. Taken together, the proximate and underlying drivers of the transitions from LFS to alternative land uses, especially intensified perennial and annual cash cropping, led to significant declines in pre-existing livelihood security and the ecosystem services supporting this security. Our results suggest that policies imposing land-use transitions on upland farmers so as to improve livelihoods and environments have been misguided; in the context of varied land uses, swidden agriculture can support livelihoods and ecosystem services that will help buffer the impacts of climate change in Southeast Asia.

**Electronic supplementary material:**

The online version of this article (doi:10.1007/s13280-016-0836-z) contains supplementary material, which is available to authorised users.

## Introduction

Global economic change has been driving the agrarian transition in Southeast Asia—a process by which regional and national economies move from being predominantly agricultural and rural to predominantly industrial, commercial, and urban (Tomich et al. 1995; Hall et al. 2011). The underlying drivers of this broad transition include the growth of state power, increasing regulation of land and forest use, population increase, and the spread of markets and infrastructure that penetrate the region’s few remaining frontiers; all of which combine to make agriculture, labour, and land-based resources increasingly market-oriented and regionally interconnected (Hall et al. 2011; Castella et al. 2013; Fox et al. 2014). These processes of change have been underway for some time (Byerlee 2014), and previous research has investigated their reworking of rural landscapes, livelihoods, and social relations, inducing smallholders to shift from food crop farming for consumption to a diversified mix of market-oriented production, off-farm wage labour, and non-farm livelihood activities (Macours and Swinnen 2002). Some writers have identified the ‘end of the peasantry’ and ‘de-agrarianisation’ as the defining features of the current transformation of rural livelihoods (Bryceson 1996; de Koninck and Ahmat 2012; Rigg 2013). In addition, more overtly coercive processes of ‘territorialisation’, ‘enclosure’, and ‘exclusion’ associated with state development and conservation have added to the complexity of agrarian change (Vandergeest and Peluso 1995; Lund 2011).

Among various land uses, swidden agriculture has been significantly impacted by agrarian change in upland Southeast Asia. Swidden (or shifting cultivation) and other forest uses continue on relatively large areas (Schmidt-Vogt et al. 2009) and support between 15 and 35 million people in the uplands (Cramb 2007; Mertz et al. 2009b). It has for centuries been integral to local livelihoods and long part of certain forest environments, integrating production from cultivated fields and secondary forests, forming the basis of land uses and customs in the uplands (Conklin 1961; Cairns 2007). As traditionally practised, swidden involves the intermittent clearing and burning of small patches of forest for subsistence food crop production, followed by longer periods of fallow in which forest regrowth restores productivity to the land (Cramb et al. 2009). A broader working definition of swidden used in this paper is a land-use system that includes a natural or improved fallow phase sufficiently long to be dominated by woody vegetation, which is then cleared and burned, to permit a shorter cultivation phase of annual crops (Mertz et al. 2009a). Traditionally long-fallow swidden (LFS) systems have often had a fallowing phase of more than 10 years, but in the last few decades, this has been more frequently reported at around 5–10 years. Hence in this review, we agreed that most studies gave fallow lengths falling within this range, while short fallow was considered to be less than 5 years. From this, we agreed upon a fallow threshold: long-fallow swiddens would be equal to or greater than 5 years, and short fallows would be less than 5 years. This fallow threshold allowed for more literature with comparative data to be included in the review. By defining swidden long fallow in the way we have, the screening criteria became much more inclusive and increased the sample of comparative datasets significantly more than if we had adopted a more stringent definition such as 10 or more years.

At the landscape scale, sustained LFS systems have given rise to and maintain complex assemblages of vegetation, ranging from open-canopy tree associations to mature closed-canopy forest (Fox et al. 2013, p. 9). As a complex system of crops and forest, LFS farming can yield various ecosystem services and resources integral to local livelihoods and forest environments in Southeast Asia’s uplands. Some suggest that swidden fallow systems constitute ‘living landscapes’, where multi-functional connections between land, forest, and farm support human wellbeing and ecosystem services (Fox et al. 2014). Mounting international policy debates, for example, at the UN-FCCC Conference of the Parties (COP) 21 concerning Reducing Emissions from Deforestation and forest Degradation (REDD+), have discussed the role of mixed forest landscapes in providing ecosystem services (e.g. carbon sequestration) and supporting livelihoods, including from variegated smallholder land uses, such as swidden and agroforestry (Mertz et al. 2012; Fox et al. 2013).

Various studies have shown how LFS can be carbon-neutral or even positive when compared to monocropped tree-based plantations (Bruun et al. 2009; Ziegler et al. 2012; Yuen et al. 2013); maintain positive hydrological properties (Ziegler et al. 2009a) across landscape mosaics over time compared to intensified land uses; reduce surface soil erosion in ways similar to intact forest in the fallow phase, and more so than oil palm plantations (Bruijnzeel 2004; Sidle et al. 2006; Ziegler et al. 2007; de Neergaard et al. 2008; Valentin et al. 2008); and support efficient soil nutrient cycling (Bruun et al. 2006).

Despite the growing recognition of LFS’s role in supporting ecosystem services and livelihood security, most international, regional, and national policies, particularly in Southeast Asia, continue to press for the replacement of swidden with other land uses (Mertz and Bruun 2016). In most rural policy discourse, swidden agriculture is still considered to be destructive to forests and to result in higher greenhouse gas emissions than other land uses (Ziegler et al. 2012), whereas monocrops of rubber, oil palm, and teak are considered to be better for carbon and livelihood outcomes, even in the absence of empirical evidence (Fox et al. 2014). These policy and socio-economic pressures are currently undermining smallholder farmers’ productive capacity and biophysical setting to deliver environmental benefits.^1^

Importantly, while these socio-economic and landscape changes intensify in Southeast Asia, there have been too few systematic analyses of the underlying and proximate drivers affecting LFS, the ability of smallholders to adapt to them, and the various outcomes that ensue in upland regions. Few studies have examined cases involving transitions from one state to another across the main socio-economic and biophysical attributes that swidden farmers negotiate in the face of profound agrarian change. While there have been many individual studies, this lack of comprehensive, integrated analysis means that there is limited conclusive empirical evidence to inform national debates and current land-use policies. This study therefore presents the first systematic review of the linkages between the drivers, impacts, and outcomes of the various transitions from LFS to other land uses over the last 25 years in Southeast Asia. These results are discussed in terms of the regional implications of these changes for globally important livelihoods and ecosystem services and climate change mitigation policies.

### Building on and going beyond previous studies

Over the last 30 years, research has shown that complex swidden fallows can support sustainable forest management, the conservation of agro-biodiversity, and relatively high labour productivity (Dove 1983; Cramb 1993; Brookfield and Padoch 1994; Brookfield and Stocking 1999). Recently, Padoch and Sunderland (2013) have argued that swidden practice reflects an example of ‘land sharing’ that can deliver both conservation and livelihood benefits across landscapes. However, governments, practitioners, and other scientists have invested heavily in a ‘land sparing’ rationale that aims to restrict less intensive land uses, concentrate and intensify agricultural production, and curtail access to land through zoning to save ‘more valuable’ forest land (Phalan et al. 2011). These land-use policies are associated with political efforts to bring remote populations under central control, replace swidden with intensified cash cropping, and provide greater access to social services (Lestrelin et al. 2012).

A number of reviews have investigated changes in swidden extent and practice across Southeast Asia (Cramb et al. 2009; Mertz et al. 2009a, 2013; Schmidt-Vogt et al. 2009) and globally (van Vliet et al. 2012). These studies have shown a trend towards swidden decline in favour of various intensified land uses and/or a move away from agriculture altogether, identifying the major drivers of swidden transitions as governance, markets, population pressure, and land access. Van Vliet et al. (2012) showed that the socio-economic and environmental outcomes of transitions away from swidden were mainly negative. Our review builds on and goes beyond these studies by empirically examining the links between specific drivers and outcomes of these transitions. It offers a comparative analysis of the social and biophysical factors driving swidden transitions and examines the impacts and outcomes of these transitions across insular and mainland Southeast Asia.

An exploration of these links is needed to better understand how *varied combinations* of drivers and transitions result in different outcomes to better inform policy decisions for the uplands of Southeast Asian. Our review therefore examines not only the major underlying and proximate drivers of transitions but also establishes the main linkages between drivers, impacts, and outcomes. We consider the drivers of emerging transitions (particularly intensified systems, cropping rotations, and protected forests) and how these impact on (1) smallholder livelihoods and (2) key ecosystem services (relating to soil properties and below- and aboveground soil carbon) previously supported by swidden in upland Southeast Asia. In so doing, we aim to answer the following question: *How do the drivers of transitions from long-fallow swidden systems to alternative land-uses impact upon livelihoods and ecosystem services in the uplands of Southeast Asia?*

## Materials and methods

The methodology below follows the systematic review protocol in Dressler et al. (2015), so only the core methodology is briefly summarised here.

### Search strategy

A comprehensive search of online academic and institutional databases as well as general search engines (e.g. Google) was conducted using terms applied in different combinations with Boolean operators. In total, 46 unique online searches were conducted using variations of search terms and strings depending on the database being searched. A full list of search terms used for each database can be found in Table S1 of the Electronic Supplementary Material.

All the returned items were checked for duplicates and saved using reference management software.^2^ In addition to the online searches, a grey literature search of relevant organisational websites and institutional libraries (e.g. Asian Development Bank, University of Philippines Los Baños) was conducted. A call for literature was also circulated via relevant email groups, list serves, and websites (see Table S1).

### Screening and inclusion–exclusion criteria

Inclusion–exclusion criteria were used to screen all articles at the title and abstract level. Only studies presenting results on changes in livelihood outcomes and ecosystem services due to transitions from long-fallow swidden (LFS) systems in the Southeast Asian uplands were included in the review. Crucially, studies had to include an in-study ‘comparator’ that compared changes from swidden to other land uses. This aspect of the systematic review methodology necessarily limited the number of otherwise relevant studies included for critical appraisal, data extraction, and final analysis.

The titles identified during the initial search phase were divided among three reviewers and screened using a bespoke screening tool (DateX). A Fleiss’ Kappa (*k*) Test (Fleiss 1971) was applied at title and abstract levels on 500 and 200 randomly selected articles, respectively. The Kappa test was repeated, with discrepancies discussed among four review team members until a minimum score of 0.6 was reached.^3^

After title and abstract screening, 795 studies were remained for full review. However, given the high number (and limited resources), the reviewers reapplied the screening criteria more strictly than before, further reducing the total number of articles for full text review to 391.

### Critical appraisal and data extraction

Four reviewers followed a standardised critical appraisal process as described in detail in the review protocol (see Dressler et al. 2015). Specifically, studies included in the final analysis and synthesis met a minimum quality standard as established in the critical appraisal process. Given this review has considered two sets of outcomes (livelihoods and ecosystem services), and the studies included a range of designs and methodological approaches, from quantitative studies with experimental designs through to observational studies and ethnographic work. In order to accommodate this diversity, we adopted a principle-based approach to critically appraise and assess the quality of studies, organised under different quality domains, such as study directness, conceptual framing, transparency, validity, and cogency among others (see Table S3). This approach allowed us to accommodate qualitative studies and methods (including non-randomised, ethnographic, and observational studies), which may not normally have been considered in more conventional systematic reviews.

Whether qualitative or quantitative, each study was initially assessed using the critical appraisal framework with a score allocated for each of the 12 questions or principles. The scores then assigned ratings of very low-to-high quality for each study (see Table S3). This was the final assessment for qualitative studies, but an additional Risk of Bias (RoB) tool (Table S4) was applied to quantitative studies (see Bilotta et al. 2014). This tool builds on the best practices examples offered by the Cochrane Collaboration and is adapted for use in environmental and natural resources reviews.

Studies deemed to have a final quality rating of low or very low were excluded from the analysis phase and archived. Studies with a quality rating of high or moderate were included in the final analysis. All components of the quality assessment were conducted for each study by at least two reviewers to minimise reviewer subjectivity. Any discrepancies were then discussed among the reviewers, and agreement was reached on the final quality assessment.

During the data extraction process, the same reviewers recorded the basic study details, and more detailed outcome data with a random sample of studies used to check the consistency of data recording. In cases where a paper reported results from more than one study site, these were considered as separate studies and assigned a unique reference number (UNID).

### Data synthesis

Data synthesis deviates the most from the approach described in the protocol (Dressler et al. 2015), and so is described in detail here. Initially, the sustainable livelihoods framework was proposed as a means of understanding any reported change in outcomes by assigning a value of between 0 and 2 to each study depending on whether the results suggested a move towards livelihood vulnerability (0) or security (2) (see Dressler et al. 2015). However, this approach was unfeasible because many studies did not include the sufficient range of livelihood capitals. Instead, individual studies were examined in terms of the described outcomes and organised into a matrix further explained in “Qualitative data analysis” section.

Quantitative ecosystem services data were synthesised using a more formal statistical meta-analysis approach. The major deviation from the protocol was that not all the anticipated ecosystem service groups or associated parameters (e.g. erosion rates and runoff) were sufficiently reported on a *comparative basis* in the studies to permit inclusion. Instead, the most robust comparative data reported on soil and carbon properties and their specific state or stock variables [e.g. cation-exchange capacity (CEC), bulk density (BD), soil organic carbon (SOC), and above ground carbon (AGC), respectively] were used rather than the flow or provision of ecosystem services.

### Qualitative data analysis

#### Driver-outcome matrix

Textual, qualitative evidence was assigned to categories in a matrix clustered into three main groups: underlying drivers, proximate drivers, and outcomes (Table S5). A binary score was assigned for each study under each category, where ‘1’ indicates the presence of drivers or outcomes and ‘0’ indicates its absence. Each study was also assigned to one of four transition typologies:

- Long-fallow swidden (LFS) to short-fallow swidden (SFS)^4^;
- LFS to perennial cash crop;
- LFS to annual cash crop;
- LFS to forest.

The specific type of transition was also captured under each typology, for example, LFS to oil palm, LFS to maize, or LFS to SFS (e.g. 15-Year Fallow to 5-Year Fallow). Given the large number of categories, the summary data presented here only represent results for those categories with total frequencies greater than the calculated median value.

### Qualitative comparative analysis

Using data from the driver-outcome matrix and frequency data, the categories with a total score greater than the median value were examined using qualitative comparative analysis (QCA).^5^ QCA uses Boolean logic to statistically conduct cross-study comparisons in a Truth Table (Legewie 2013), which determines whether certain factors (conditions or drivers) or combinations thereof produce a given outcome and in how many cases. The QCA was only applied to the textual, qualitative data of the livelihoods analysis; the quantitative data were examined separately in a formal meta-analysis (“Qualitative comparative analysis” section).

We used QCA to analyse each of the three most common underlying and proximate drivers and the full list of outcomes under all land-use transitions. This process was then repeated for each transition individually, using the results for those studies reporting a transition to annual cash crops, perennial cash crops, or shorter fallows.

### Quantitative data: Meta-analysis

The majority of quantitative data were extracted from studies reporting soil properties and aboveground carbon stocks. As such, statistical meta-analysis was only possible for these studies. For parameters reporting results using different units, the standardised mean difference (SMD) or ‘Hedge’s *g*’ was selected to permit comparison (Higgins and Green 2011). In cases where the units were the same or could be easily and accurately converted, the continuous mean difference was applied instead. It was assumed that because a range of data collection methods were used in the studies and that the subject groups (agricultural systems in a variety of contexts) would vary; there would be real differences in reported results or effect sizes. For this reason, all meta-analyses were run as random effect models as opposed to fixed effect models, which assume a single ‘true’ effect size (Higgins and Green 2011).

Data relating to a variety of ecosystem service domains and associated parameters were compiled in a matrix and organised by transition with values for LFS compared with those for alternative land use. Soil properties for which there was sufficient data to permit meta-analysis included SOC, BD, and CEC. The only ecosystem property with sufficient data for meta-analysis was aboveground carbon stock (AGC). Studies included for analysis had to report mean, standard deviation, and sample size for a given parameter. Scholars were contacted to request any missing data or to estimate standard deviation from the standard error and sample size if available. In some instances, data were extracted from published figures using an online application, Web Plot Digitizer.^6^ When this information was irretrievable, the study was excluded from analysis. Each of the parameters was then treated using Open Meta Analyst.^7^

Meta-analysis was conducted on all data points for a given parameter regardless of transition type to assess the overall effect of a shift from LFS to any alternative land use. This was performed separately for soils data in the upper horizon (up to a depth of 10 cm) and for data in the lower horizons (20–40 cm). Subsequently, individual meta-analyses were conducted for each transition type for each parameter, again at two different soil depths. The results were presented numerically and graphically as a forest plot^8^ (see Figs. 3, 4, 5, 6). Tests for heterogeneity included *χ* ^2^, degrees of freedom, *p* values, and *I* ^2^, and were reported for all analyses. Results were considered significant at the 90 % level and *I* ^2^ values of 75 % or below were considered acceptable (Higgins et al. 2003).

### Effect modifiers

Where reported in the studies, potential effect modifiers or confounding variables were recorded. These are variables that have the potential to influence the outcomes reported and which may limit the ability to generalise findings. They included topography, forest ecosystem type, elevation, pedological factors (e.g. soil type and texture), application of inorganic inputs (e.g. fertiliser, herbicide, and pesticide), climate (e.g. rainfall and temperature), and slope.

## Results

### Search, screening, and critical appraisal results

Online searches returned a total of 17 198 articles supplemented by 11 articles from organisational websites, 62 articles from direct searches in libraries, and 45 articles suggested by experts in the field. A grand total of 17 208 unique journal articles, book chapters, project reports, theses, and conference proceedings were found and stored after the removal of duplicates. Table 1 summarises the results of various stages of screening and critical appraisal. In total, data were extracted from 93 different study sites across 82 different papers (a complete list can be found in Table S2).

**Table 1:** Summary of screening, critical appraisal, and data extraction process with remaining studies at each stage

Review Stage	Action
Full title search	*n* = 17 208 titles returned
Title screening	*n* = 3051 relevant titles retained
Abstract screening	*n* = 391 relevant abstracts retained
Critical appraisal	*n* = 110 retrievable studies of sufficient quality (*177 Excluded, 10 duplicates removed, 82 missing texts, 2 very low quality, 22 low quality, 80 moderate quality and 18 high quality studies*)
Data extraction	*n* = 110 articles reduced to 93 separate studies

### Description of included studies

**Fig. 1:**
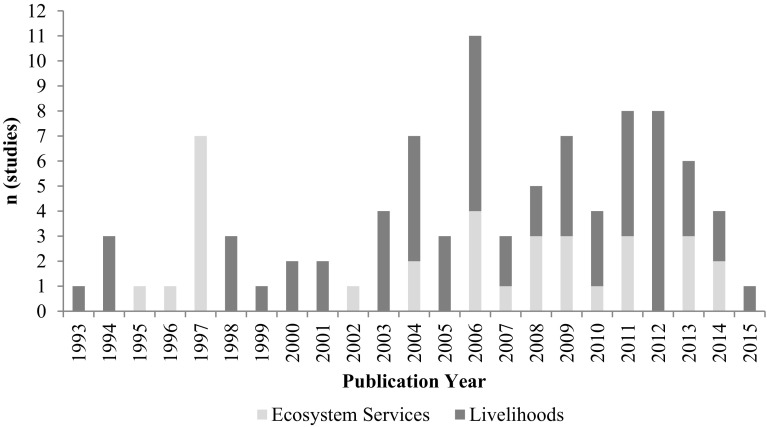
Summary of temporal distribution of studies, organised by outcome area

The majority of studies came from peer-reviewed journals, published between 1993 and 2015 (Fig. 1).

Of the 93 different studies, the majority (60) were focused on livelihood outcomes, most of which (39) used qualitative methods. There were 33 studies presenting results for ecosystem services, with most using quantitative methods. Only three used mixed methods and one was qualitatively oriented.

**Fig. 2:**
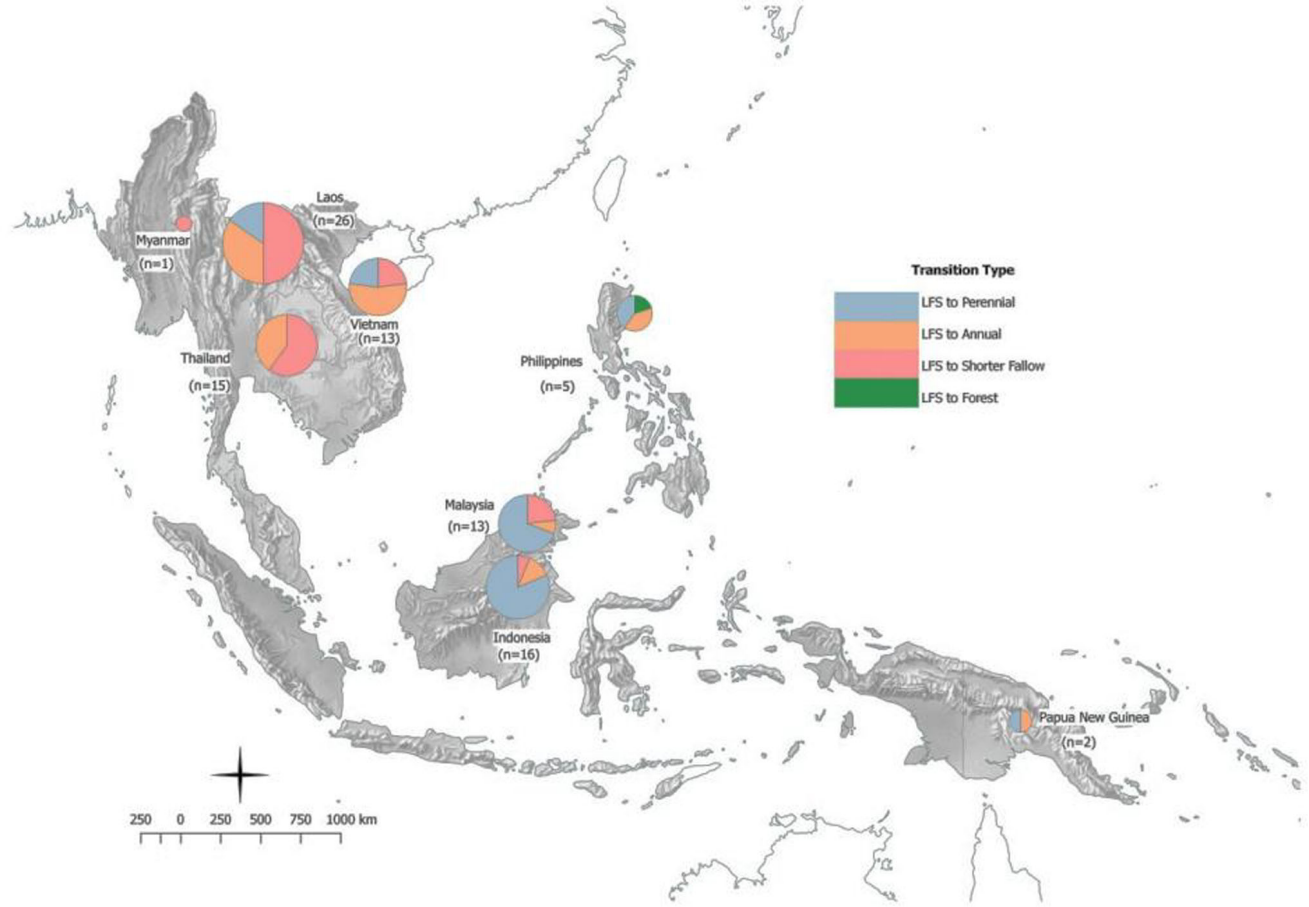
Number of studies reported by country and transition type. The *pie charts* indicate the proportion of studies for each transition type with the total number of studies (*n*) shown in *parentheses*. The size of each *pie chart* is relative to the number of studies i.e. larger pie = more studies

The studies covered a wide geographical distribution. Most studies were located in Laos (*n* = 26), possibly reflecting the persistence (and recent rise in study) of swidden and associated transitions there compared to elsewhere. The majority of the remaining studies were distributed between Thailand, Vietnam, Malaysia, and Indonesia with 5 studies from the Philippines, 2 in Papua New Guinea, and 1 in Myanmar (Fig. 2). The authors also defined the distribution of studies into four transition types drawn from grouping the study comparators from the selected literature (see also Fig. 2). These include a clear trend for transition from LFS to shorter fallows or annual crops in mainland Southeast Asia (Vietnam, Laos, Thailand and Myanmar); a transition to perennial crops in northern Laos and Vietnam (in most cases to rubber, see Ziegler et al. 2009b; Fox et al. 2012); and a transition to perennial crops, including, but not limited to, oil palm and rubber plantations in insular Southeast Asia (Malaysia and Indonesia). At the regional level, 33 studies (36 %) describe transitions to perennial crops, 31 studies (33 %) transitions to shorter fallow systems, 28 (30 %) transitions to annual crops, and just one study (~1 %) investigates a transition to permanent forest cover.

### Drivers of land-use transitions and livelihood outcomes in Southeast Asia

This section describes the results for underlying and proximate drivers of swidden transitions and their livelihood outcomes.^9^ These results are based on qualitative livelihoods data and disaggregated by transition type.

#### Underlying drivers

The major underlying drivers of the transition from LFS to alternative land uses documented in the included studies were land-use policies, population pressure, and market influences. We characterise land-use policies as purposive (state, bilateral, and NGO) policies determining and influencing the governance, access to, and use of land (for example, forest and land allocation, conservation reserves, and agricultural zones). Population pressure in upland areas refers to any association of endogenous, demographic growth and increases due to in-migration or (re)settlement within swidden and non-swidden communities. These population-increase dynamics can be driven by lowlander and uplander responses to perceived economic opportunities and/or enforced through compulsory government resettlement schemes. Market influence broadly refers to the increased economic exchange of commodities (typically from forests and agriculture) based on monetary values. Our definition also includes sub-themes such as access to markets via intermediaries, increased prices for products, and incentive schemes from national governments or other actors to increase the production of certain crops.

Of these major drivers, land-use policies arose most frequently in the review (59 times) as the most important driver of change overall. This was also disaggregated across each transition type from LFS to annual crops (23), perennial crops (19), and short fallow systems (16). Market influence was the most important driver in terms of the shift from LFS to perennial crops. This indicates the influence that existing and newly emerging markets have in inducing farmers to plant perennial crops (e.g. rubber, oil palm, coffee, and timber) within swidden systems for sale through rural traders, direct buyers, or transportation to markets or processors, as well as the land-use policies that support these market transitions. After land-use policies, population increase was the next most important factor inducing the shift from long-fallow to short-fallow systems (15) (Table 2).

**Table 2:** Summary table showing the number of studies citing major underlying and proximate drivers, organised by the type of transition from Long-Fallow Swidden (LFS) in Southeast Asia

Transition	Underlying drivers			Proximate drivers		
	Land-use policies	Population increase	Market influence	Individualised tenure	Intensification	Restricted land access
LFS to forest	1	0	1	1	0	1
LFS to annual crops	23	9	15	10	19	14
LFS to perennial crops	19	7	30	13	24	12
LFS to shorter fallow	16	15	8	8	18	10
Grand total	59	31	54	32	61	37

#### Proximate drivers

The most significant proximate drivers of the transition from LFS to a range of other land uses included (from greater to lesser importance) intensification, restricted access to land and individualised tenure. Land-use intensification generally referred to the process by which upland swidden farmers increased production from a fixed or reduced area of land. In these studies, LFS or the alternative land uses were intensified primarily as a result of policies which restricted, altered, or influenced the type or extent of swidden practice. Restricted access to land referred to the process of physically restricting access to land via enforced allocation, enclosure, or imposed tenure arrangements. Individualisation of tenure referred to instances where customary land rights gave way to private (de facto or de jure) ownership of land (by either an individual or family), which drove transitions away from (more extensive) traditional uses and often towards activities increasing household income and benefits. Some studies, however, described circumstances in which this could be as much an outcome as a driver (e.g., smallholders planting cacao on shared fallow lands to remove it from the commons and individualise tenure: see Belsky and Siebert 2003; Feintrenie et al. 2010).

The process of intensification was a significant direct driver (cited in 61 studies) of the transition from LFS to other land uses, particularly permanent perennial crops (24)—a transition that was indicative of producing more (cash) crops on fixed or smaller plots of land. Restricted access to land and individualised tenure were frequently cited direct drivers of shifts from LFS to permanent annual crops (14) and permanent perennial crops (13), respectively. Restricted access to land and individualised tenure both facilitated transitions to shorter fallow periods (10 and 8 studies, respectively). No significant land access restrictions existed in cases where LFS returned to mature forest cover.

#### Livelihood outcomes

The overall influences of underlying and proximate drivers of the main transitions are linked to a range of mainly negative outcomes for the livelihoods of uplanders in Southeast Asia. The most widely reported, illustrative livelihood outcomes included *reduced access to swidden fallow* (41) and *a decline in staple yield* (33) due to (a perceived decline in soil fertility from) shifts to permanent annual and perennial crops, and a rise in short fallow systems (Table 3). These outcomes are clearly related and cannot be treated in isolation, as shown in the quotations below.

**Table 3:** Summary of studies citing major livelihood outcomes as a result of transitions away from long-fallow swidden (LFS) in Southeast Asia

Transition	Outcomes							
	Reduced access to fallow	Decline in staple yield	Reduced socio-cult. wellbeing	Narrowed LH option	Deprioritisation upland rice	Increase labour input	Reduced customary practice	Increased income
LFS to forest	1	0	1	1	0	0	1	0
LFS to annual crops	12	11	8	10	9	10	10	8
LFS to perennial crops	17	13	10	12	15	11	7	12
LFS to short fallow	11	9	3	4	3	5	2	0
Grand total	41	33	22	27	27	26	20	20

These two main outcomes contributed to food insecurity through changes in access and productivity of land. The following text evidence exemplifies the role of land-use policies in influencing access to fallow land. Several studies, including Castella et al. (2013) and Jakobsen (2006), show how land-use policy influences access to fallow lands and induces transitions. Castella et al. (2013) write, for example, that in the Lao PDR, the “*LUPLA [Land Use Planning and Land Allocation Policy] also aimed at substituting individual land certificates for customary land tenure arrangements (i.e. right of clearance or ‘axe rights’). Each household was allocated three upland plots, which meant that the maximum fallow period possible was limited to two years*” (Castella et al. 2013). Jakobsen (2006) notes for Vietnam how “*following the restrictions on shifting cultivation, the total area of fallow land also began decreasing, while the area of secondary forest began increasing*” (p. 107).

In terms of declines in staple yield, Lestrelin et al. (2012) show how reduced fallow systems have produced “*decreasing yields due to impoverished soil and land lost to gullies*” (p. 69). Similarly, Ducourtieux (2006) finds that “*Land allocation has had a direct impact on swidden cultivation in Yapong. The forest reserves are taken out of rotation, then the surface area of fallow land available for swidden cultivation regressed. The age of the fallow upon slashing dropped from 10 to 3* *years. Yield is limited to 600* *kg/ha of paddy rice on the plot cleared in the year, compared to 1300* *kg/ha for the village of Samlang, in a forest zone, i.e. a 54* *% reduction*” (p. 17).

Typically associated with these outcomes was a range of adverse impacts, including a *narrowing of livelihood options* (27) and the *deprioritization of upland rice* (27), which tend to reduce socio-economic ‘buffers’ to food insecurity. These were often associated with *reduced socio*-*cultural well* *being* (22) and *reduced customary practices* (ritual, etc.) (20). While some studies indicated an *increase in overall income* from the shift from swidden to permanent perennial crops (12), which stands to benefit household finances and may offset a decline in staple yield, there was broader indication of constraints being placed on the foundations of household livelihoods. This includes, for example, an *increase in labour input* (26) and *income* (20) from investing in perennial crops and annuals that come at the expense of overall social and cultural wellbeing (Table 3). In time, however, many farmers’ socio-economic standing may rise as they consciously invest more capital into cash cropping for greater financial return.

### Qualitative comparative analysis results

In terms of underlying drivers, the combination of land-use policies and markets influenced a third of the cases involving a transition to annual crops and decline in staple yields (Table 4). This same combination of factors was also present in 76 % of cases in which access to fallow land was reduced under a transition to perennial cash crops. In almost half of the cases, land-use policies alone (without the influence of markets and population increase) contributed to a reduction in staple yield (44 %) and reduced access to fallow land (45 %) under short-fallow transitions. In most cases, then, land-use policies worked somewhat independently to strongly influence a range of outcomes.

**Table 4:** Summary results of qualitative comparative analysis (QCA). Percentage figures represent the proportion of studies whose reported outcome can be explained by that combination of drivers with absolute number of cases in parentheses. Note that only statistically significant results (i.e. those with a consistency score of ≥0.7 and coverage scores of ≥25 %) are included here. Blanks in the table indicate results for the combination of drivers for that outcome were not statistically significant and therefore not included

Combination of drivers by transition type	Outcomes							
	Decline in staple yield	Deprioritisation of upland rice	Increase in labour input	Increased income	Narrowed livelihood options	Reduced access to fallow land	Reduction in customary practices	Reduced Socio-cultural wellbeing
All transitions								
Individualisation of tenure + restricted access to land					52 % (19)			
Intensification + restricted access to land						80 % (20)		
LFS to annual cash crops								
Absence of individualisation of tenure + restricted access to land	45 % (6)							
Individualisation of tenure + intensification			60 % (7)		60 % (7)		50 % (7)	63 % (8)
Individualisation of tenure + restricted access to land						58 % (8)		
Intensification + absence of restricted access to land				50 % (8)				
Land-use policies + market influences		33 % (4)						
Population increase + absence of market influence	27 % (3)					25% (3)		
LFS to perennial cash crops								
Individualisation of tenure + intensification			64 % (8)					
Individualisation of tenure + restricted access to land	46 % (8)	53 % (5)			60 % (8)	71 % (8)		
Land-use policies + population increase					25 % (3)			
Land-use policies + market influences						76 % (16)		
LFS to shorter fallow								
Absence of land-use policies + population increase								
Individualisation of tenure + restricted access to land						73 % (9)		
Individualisation of tenure + absence of intensification	33 % (2)							
Land-use polices + absence of market influence	44 % (5)							
Land-use policies + absence of population increase						45 % (5)		

Across all transition types, 80 % of cases documenting reduced access to fallow land involved a combination of land-use intensification and restricted access to land. Under transitions to annual cash crops, the combination of individualisation of tenure and land-use intensification was the most influential mix of proximate drivers. In combination, they affected more than half of the cases (between 50 and 63 %, with statistical significance) across 4 of 9 outcome areas including an increase in labour input, narrowed livelihood options, reduction in customary practices, and reduced socio-cultural wellbeing.

As combined proximate drivers, the individualisation of tenure and restricted access to land were present in 46 % of cases observing a reduction in staple crop yield; 53 % of cases in which upland rice was deprioritised; 60 % of cases in which livelihood options were narrowed; and 71 % of cases in which access to fallow land was restricted. Similarly, this combination of proximate drivers also explained 73 % of cases involving a reduction in access to fallow land under a transition to shorter fallow systems.

### Land-use transition impacts on ecosystem services: Soil properties and aboveground carbon stocks

Land-use transitions were also examined in terms of their impact on soil properties and aboveground carbon (AGC) stocks in swidden fields themselves; these serve as key components of soil fertility and carbon sequestration, two important ecosystem services. We broadly characterise ‘ecosystem services’ as the beneficial biophysical components of nature that are used to support peoples’ wellbeing and livelihoods, and the overall resilience of forest landscapes (Boyd and Banzhaf 2007).

**Fig. 3:**
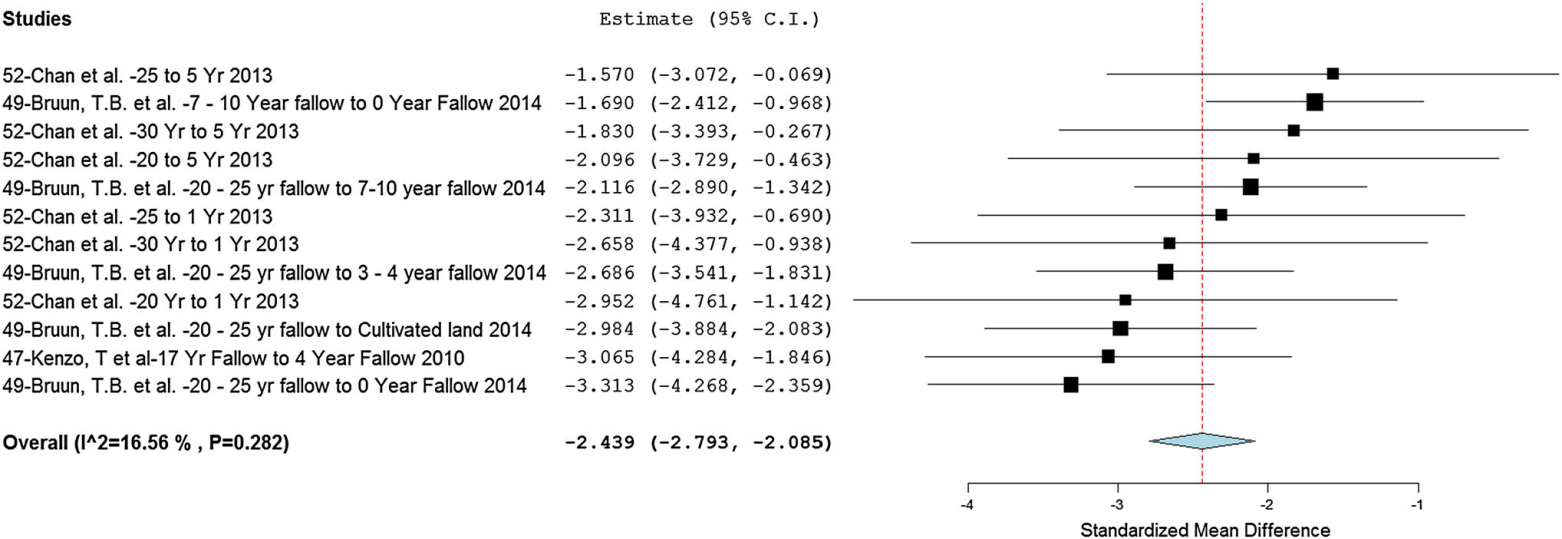
Forest plot showing the effect of transitions from long-fallow swidden to other land uses (mainly changes in fallow length) on aboveground carbon stock (mixed units)

We examined the impact of transitions from swidden to other land uses on soil properties and AGC by conducting a quantitative meta-analysis using 29 of the 93 studies included in the review. These studies used one of three methodologies to compare impacts: (1) cross-site comparisons; (2) substitution of space for time (the chronosequence approach); or 3) ‘before and after’ land-use change studies. Results are presented for those significant at the 95% level and with a low-to-moderate level of heterogeneity of ≤75 % (Higgins et al. 2003).^10^ The transition from LFS to short or no fallow (continuously cultivated) systems revealed an overall substantial negative effect (−2.439) on AGC (Fig. 3).^11^ Results show low heterogeneity across the reported study results (*I* ^2^ = 16.56 %), which suggests that over 80 % of the variability could be owing to non-random treatments effects. Taken together these results indicate that there was an overall negative effect on AGC as land-use transitions from longer to shorter fallows.

**Fig. 4:**
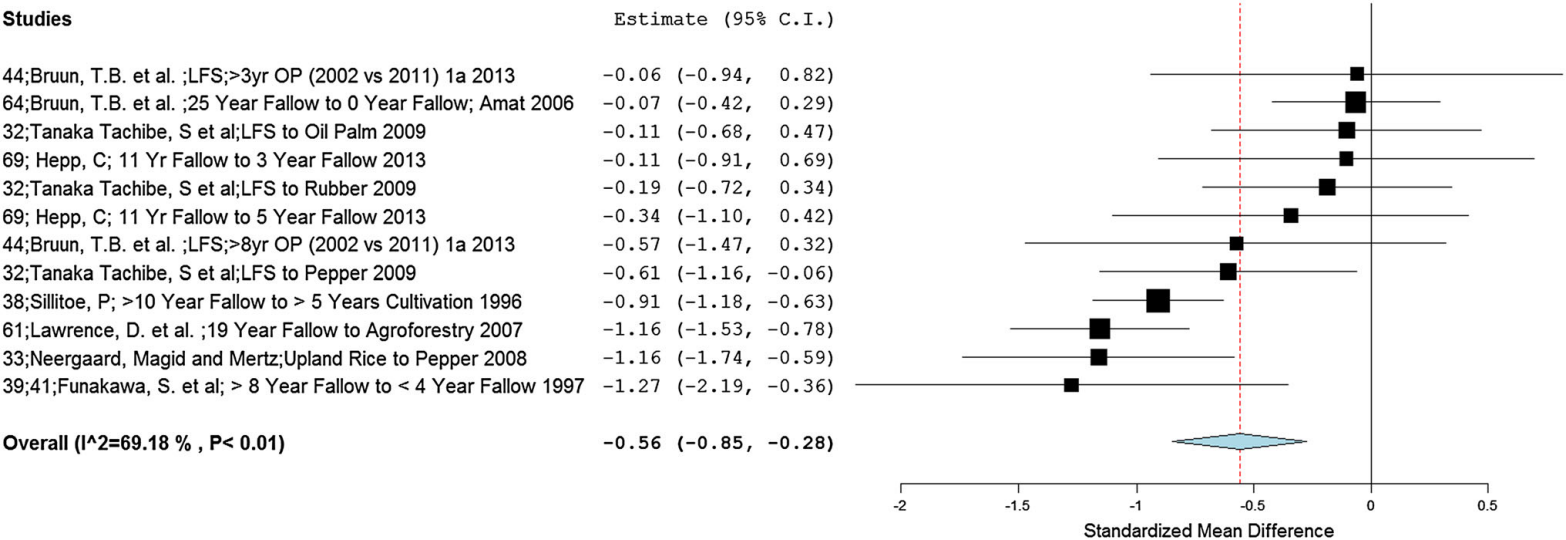
Forest plot showing effect sizes and summary effect size for concentration of soil organic carbon (%) across all land-use transitions at a soil depth of 0–10 cm

SOC showed an overall negative effect (−0.56) with the shift from LFS to alternative land uses resulting in reduced overall levels of soil carbon (Fig. 4). These results are significant (*p* < 0.01) and have a moderate level of heterogeneity (*I* ^2^ = 69.18 %). As an important determinant of soil health and productive capacity, even a small reduction in SOC in the upper soil horizon can negatively affect crop productivity and overall agricultural yields (Lal 2006; Söderström et al. 2014).

**Fig. 5:**
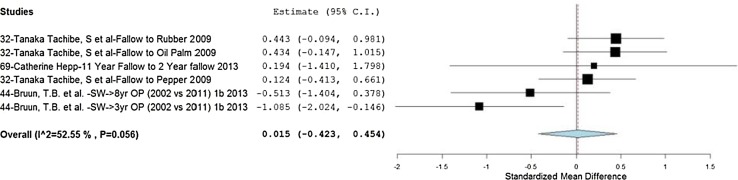
Forest plot showing effect sizes and summary effect size for soil bulk density (inverse values) across all land-use transitions at a soil depth of 20–40 cm

The only significant results meeting the heterogeneity threshold (<75 %) were those from soil depths of 20–40 cm, and there was no overall effect detected in the reported results from these 3 studies (Fig. 5).^12^ A large amount of variability can be observed in these results (−0.423 to 0.454) with a moderate level of heterogeneity (*I* ^2^ = 52.55 %) and effects just outside the 95 % significance level. This suggests that effect modifiers may have a substantial influence on BD, which may not have been captured or adjusted for in the study results. These results may also reflect the fact that soil BD is an extremely variable parameter, which tends to be under sampled given the labour intensity involved (Don et al. 2011).

**Fig. 6:**
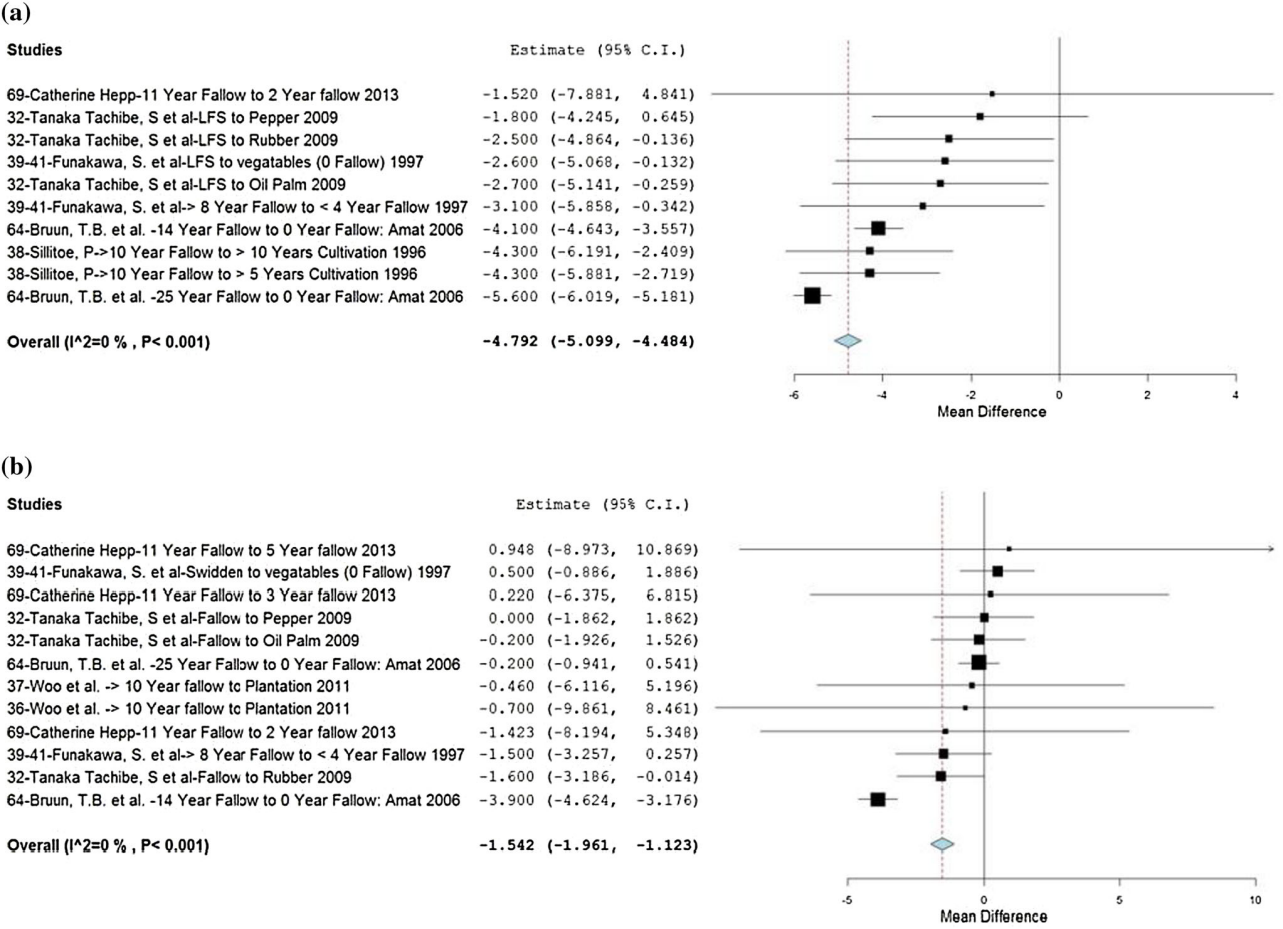
**a** Forest plot of effect sizes for cation-exchange capacity (cmol kg^−1^) at a soil depth of 0–10 cm. **b** Forest plot of effect sizes for cation-exchange capacity (cmol kg^−1^) at a soil depth of 20–40 cm

There are substantial and statistically significant reductions in CEC for both the upper (0–10 cm) and lower (20–40 cm) soil horizons associated with transitions from LFS to more intensive land uses, each with very low heterogeneity (Fig. 6a, b). In the upper soil horizon (Fig. 6a), effects are negative across all studies, with transitions to alternative land uses from LFS resulting in reduced CEC. In the deeper soil horizons (Fig. 6b), effects are more variable with a smaller overall effect size compared to that in upper horizons. This could be attributed to the influence of soil type and underlying geology which may influence CEC at lower depths, whereas in the upper 10 cm, management practice and land-use type tend to have more of an effect. As the studies are from areas with low activity clay soils (mainly Ultisols dominated by kaolinite with a very low CEC value of 2–10 cmol kg^−1^), the CEC value may be affected by the content of soil organic matter, which is also negatively affected in studies reporting both of these parameters. In general, then, land-use transitions out of swidden fallow into more intensified reduced fallow systems result in a decline in soil fertility.^13^

## Discussion

This systematic review began with the question: *How do the drivers of transitions*—*and the transitions themselves*—*from LFS systems to alternative land-uses impact upon livelihoods and ecosystem services in the uplands of Southeast Asia?* In answering this question, we examined the drivers, impacts, and outcomes of these transitions and the linkages interconnecting them. The question has long been part of forest governance policy debates concerning the relative impact of swidden systems on the forest resources and ecosystem services of economic importance to nation-states across Southeast Asia. In most cases, long-standing negative assumptions about swidden agriculture have led to its vilification and criminalisation (Conklin 1954; Kunstadter et al. 1978; Fox 1982; Dove 1983; Mertz and Bruun 2016), giving rise to punitive policies that have significantly impacted upon livelihoods and landscapes in the uplands (Fox et al. 2014)—–something Dove (1983) called the ‘political economy of ignorance’. These national policies remain remarkably resilient despite numerous studies showing that the potential remains for swidden to support poor upland peoples’ livelihoods and ecosystem services, and that swidden farmers are best placed to manage their systems at an intensity and scale appropriate to their needs (Conklin 1954; Brookfield and Padoch 1994; Zimmerer 1996; Fox et al. 2000, 2014; Cramb et al. 2009; Dressler 2009).

Swidden policies are perhaps more nuanced today than in the past, as some state and NGO policies support the livelihoods and land rights of swidden famers (Dressler and Pulhin 2010). Nevertheless, polemical policy debates concerning the impacts of swidden continue among international agencies, governments, civil society groups, and upland farmers themselves (Astuti 2015). While the decline of swidden fallow in Southeast Asia is clearly due to the above-mentioned policy interventions and other drivers, our analysis (and those of authors we have cited) demonstrates that LFS still contributes to livelihood security and ecosystem services for many poor uplanders today. In many parts of Southeast Asia, swidden farmers continue to manage their land, livelihoods, and social relations as the process of agrarian change intensifies to encompass increasing commodity production (e.g. palm oil, rubber, and teak); expanding infrastructure development (dams, mines, roads, bridges and ports); rising populations and in-migration; outmigration to engage in paid labour; and various restrictions over land access and use.

In many ways, then, the debate over the relative impact of LFS remains unresolved, contested, and ambiguous in policy arenas and development interventions (Fox et al. 2014). This review identified the key drivers affecting swidden transitions, the main transitions, and their associated impacts on livelihoods and (selected) ecosystem services in the region in order to better inform this policy debate. By focusing explicitly on swidden transitions**—**from one state to another**—**with robust, standardised data, we offer a definitive statement on the broader value of swidden practices in supporting ecosystem services and livelihoods in the uplands of Southeast Asia.

### Underlying drivers of land-use change

Confirming the global findings of van Vliet et al. (2012), our analysis of the aggregated review data found that, rather than just economic or social factors, land-use policies were the most significant underlying driver of the transition from LFS to alternative land uses in Southeast Asia (e.g. Hansen and Mertz 2006; Cramb et al. 2009). These are mainly state-defined and driven land-use policies that advocate the intensive planting of annual and perennial crops with an associated reduction in swidden fallow, or conservation policies that allocate swidden lands to protected areas (e.g. Belsky and Siebert 2003; Feintrenie et al. 2010). Fox et al. (2009) contest that land-use policies reflect a system of classification (thematic types and labels) and designations (zoning and incentives) that classify people and agriculture in terms of swidden and permanent agriculture in designated zoning schemes. In some cases, such land-use zoning tends to associate swidden with particular ethnic categories and facilitates relocation to zones wherein cash cropping is adopted and supported (Castella et al. 2013); in others, smallholders may join cash cropping schemes largely under their own volition in designated land-use zones (Fox et al. 2014).

Land-use policies and market influences often work together in affecting the extent to which swidden landscapes are converted to produce forest and or agricultural commodities for domestic consumption or trade. We showed that market influences strongly affect perennial cash crop production, particularly rubber and oil palm, in former swidden landscapes. Moreover, the QCA shows that the combination of land-use policies and markets played a significant role in influencing the transition of swidden to perennial cash crops (e.g. Evans et al. 2011). As part of this, increasing commodity prices were frequently cited signals for governments, corporations, and farmers to undertake or promote the transition from swidden fallow to monocropped plantations of oil palm, rubber, and teak, among others (Dendi et al. 2004; McCarthy 2010). Coupled with population pressure, these underlying drivers produced the ideal ‘enabling’ conditions for the conversion of swidden landscapes to short-fallow systems and permanent tree-crop or annual crop agriculture (Wadley and Mertz 2009).

### Integrated proximate drivers of land-use change

The range of proximate drivers involved a general trend toward the *intensification of land uses, individualised tenure, and restricted access to land* in transitions out of swidden. While some proximate drivers of LFS transitions can have an impact separately from others (including, for example, restricted access to land driving the shift to shorter fallows), the QCA showed that this is seldom the case and that drivers in combination elicit many of the outcomes. For example, broader constraints of access to land and patterns of intensification were most commonly associated with reduced access to fallow land in upland areas. Similarly, in cases where tenure is increasingly privatised (involving a shift from common access to individual property rights), there is greater likelihood of intensification and investment in annual crops. Once separated from the forest commons typically in the uplands, the rise in individualised tenure renders former swidden lands alienable to others through formal or informal market transactions. In contrast, in cases where swidden fallows regrow into mature forest (as usufruct rights attenuate over time and space), fewer access restrictions prevail (Dressler et al. 2012).

The livelihood and biophysical outcomes for upland farmers experiencing transitions from LFS to other land uses were overwhelmingly negative. The shift from swidden systems to permanent annual and perennial crops and the rise in short-fallow cultivation led to declines in staple yield and reduced soil fertility over time, together reinforcing household food insecurity. This was particularly the case where swidden farmers experienced a narrowing of livelihood options as they increased labour input to specialised production of one or more annual cash crops—a trend well established in certain literature (Cramb et al. 2009). While those swidden farmers who shift to cash crops experience an overall increase in household income, they also tend to deprioritise upland rice production, resulting in declining supplies in staple food crops, and experience a reduction in customary practices and socio-cultural wellbeing. As the QCA showed, those farmers working individual holdings with annual or perennial cash crops tended to do so more intensively for commodity markets, rather than produce crops for local consumption and redistribution. While such livelihood trajectories were rapidly intensifying, with market engagements often significantly increasing overall income, there was an associated loss of diverse economic activities; this outcome can increase household risk and taking time away from activities related to ‘domestic reproduction’ and socio-cultural investments. In cases where more family labour was devoted to producing commodities for sale than for domestic needs (often involving greater debt), households may experience a commodity ‘reproduction squeeze’ (Bernstein 1986). With an increase in labour input and sustained intensification, the fields supporting perennial and annual crops also suffered a decline in soil fertility related to shorter or zero fallowing.

The QCA showed, moreover, that land-use policies**—**even in the absence of other factors, namely markets and population increases**—**directly contributed to reduced access to fallow lands and declining staple yields. Importantly, LFS farmers whose staple production declined due to zoning restrictions and had limited access to markets for cash crops faced fewer livelihood opportunities and so suffered both income and food insecurity. The absence of state livelihood support, in such instances, increased livelihood risk, particularly when access to forest-fallow products (e.g. non-timber forest products) was limited and no longer provided an income buffer (Koczberski et al. 2012).

### Ecosystem properties: Soil and carbon

Our meta-analysis of quantitative data revealed trends in the effects of transitions on soil properties and carbon stocks that were consistent with those observed in the livelihoods data. Across most parameters for which there were sufficient data, significant results, and low heterogeneity, the indicators of soil fertility (i.e. SOC and CEC) and carbon stocks in LFS were negatively affected by transitions to alterative land uses. These effects were consistent across all transition types. The only exception was for BD where there was no discernible effect. Results varied with soil depth, with major impacts being observed in upper layers (in particular CEC and SOC), that are the most active in terms of nutrient cycling. However, at soil depths of 20–40 cm, roughly half of the meta-analysis results showed a negative effect on key soil properties with the remaining half showing no net effect.^14^

Thus, land-use changes from LFS to perennial or annual cash crops or short-fallow systems have net negative impacts on overall soil fertility. Over time, this decline in soil fertility negatively impacts on the growth and yield of cash crops and monoculture plantations and potentially reduces surplus production and annual income, unless production is maintained or increased by applying chemical fertilisers. However, the application of inorganic fertilisers may not support production indefinitely and could, in extreme cases, lead to an exhaustion of soils that could, in turn, limit farmers’ options to return to more labour-intensive, less capital-intensive practices. The requirement for sustained inputs to maintain productivity in intensified monocropped systems can also increase capital outlays and indebtedness among former swidden farmers with limited savings (Nguyen et al. 2004; Valentin et al. 2008).

AGC was higher in LFS than in alternative land uses, indicating the significantly reduced carbon storage capacities in more intensified, monocropped systems at the plot or field level. With the expansion of intensive perennial and annual cash cropping systems replacing swidden systems in the uplands, there will be broader negative impacts on aboveground carbon storage at the landscape scale. This supports findings from other reviews of carbon dynamics from various land uses in Southeast Asia (Bruun et al. 2009; Ziegler et al. 2012). While swidden has ‘conspicuous’ sources of CO_2_ emissions when fallow forests are cleared and burnt, much of the landscape under LFS sequesters carbon during the regenerative fallow stage. Intensifying the fallow cycles or replacing forest with annual or short rotation crops results in lower average carbon stocks over time across the landscape (see Ziegler et al. 2012). Consequently, increased CO_2_ emissions are likely to result from all of the land-use transitions identified in the study.^15^

At the landscape scale, the reduction in AGC involving transitions from LFS to more intensified land uses therefore has important implications for climate change policy. Indeed, the corollary of this is that positive carbon outcomes can be achieved with policy interventions that increase the areal extent of trees outside forests by extending fallow rotations or incorporating a forest-fallow period in crop production systems. Depending on the overall carbon dynamics at the landscape scale and the design of government subsidies and or other payment systems, mechanisms like REDD+ may provide financial benefits for local communities while allowing them to continue local food production under traditional fallow systems (van der Ploeg 2014). The prospect of managing for broader multi-functionality across upland forest—farming landscapes (see Wiggering et al. 2003) already aligns with COP 21, wherein those countries party to the agreement ought to promote integrated, sustainable forest management in support of upland farmers’ right to land, livelihood, and climate change adaptation.

**Fig. 7:**
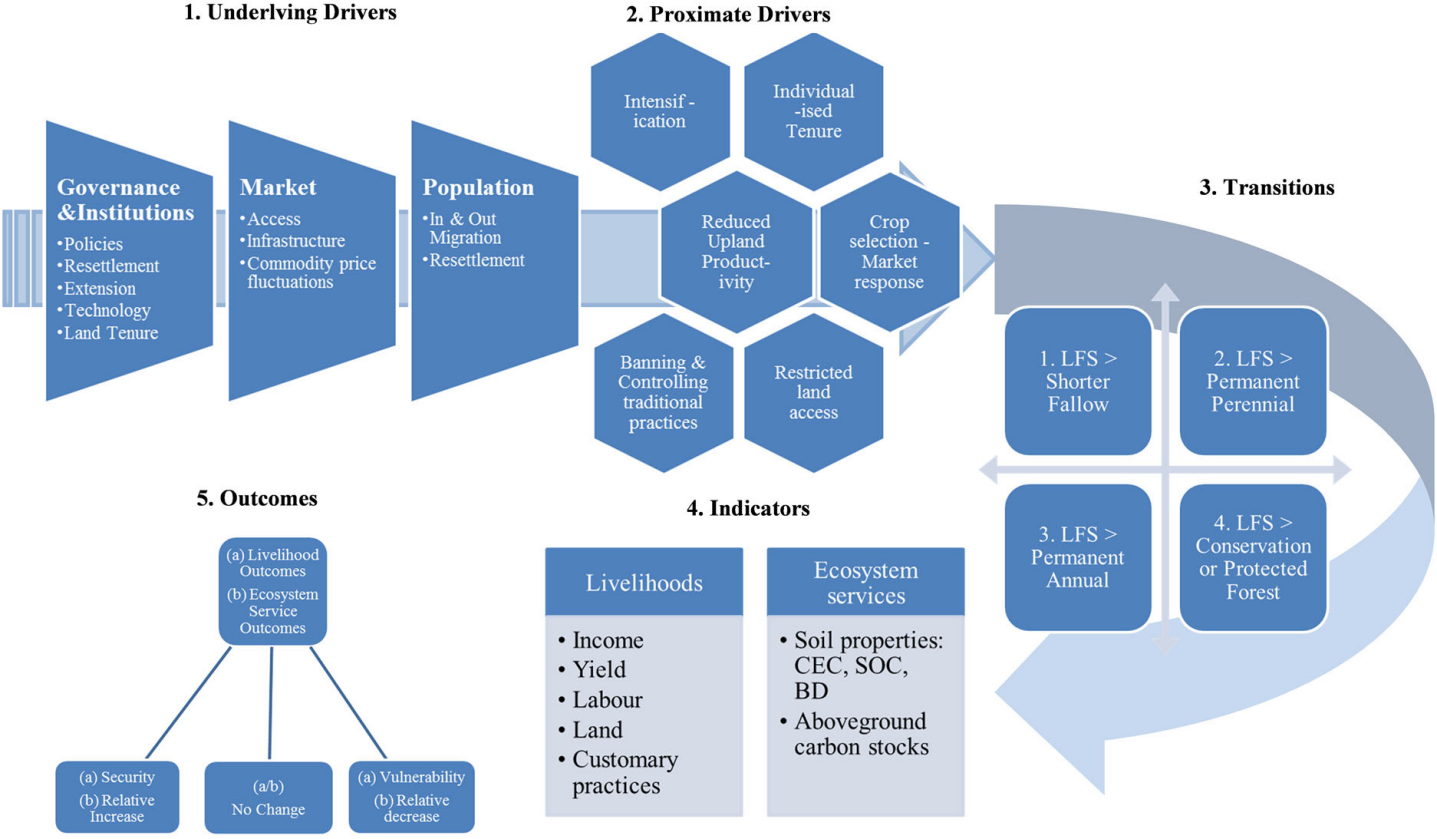
A model of the drivers and outcomes associated with transitions from long-fallow systems to alternative land uses in terms of livelihoods and ecosystem services

In summary, the evidence from this review points towards consistently negative impacts arising from transitions from LFS to other land uses across a range of livelihood and ecosystem service indicators. Such transition impacts are primarily driven through various integrated factors (land-use policies, infrastructure and market expansion, and population growth/in-migration, among others) that constrain and or eradicate LFS in the uplands (see Fig. 7). Our analysis shows a connection between underlying drivers resulting in transitions from LFS to alternative land uses and associated impacts and outcomes on livelihoods and ecosystem services. *Underlying Drivers*, including governance and institutions, markets, and population and land access issues, contribute to *Proximate Drivers* that strongly influence the type of transition in the medium term and livelihood and landscape level outcomes in the longer term. *Transitions* from LFS generally resulted in four groups of alternative land uses, including: (i) shortened fallows (intensification); (ii) LFS being replaced wholly or partially by perennial crops such as oil palm; (iii) LFS giving way to annual monocrops such as maize; and (iv) territorial enclosure of former swidden areas (for conservation, plantations, agriculture, etc.). The *Outcomes* indicate that those farmers who were once dependent on LFS-based livelihoods may have increased vulnerability, and reduced food security, or may not be affected by the transition in any significant way (no change).^16^ These results challenge current policies and practices that aim to tackle upland poverty, deforestation, and climate change by replacing LFS with more intensive and or restrictive land-use systems.

Our findings suggest that if such negative outcomes on swidden farmers and ecosystem services are to be avoided, policies and programme interventions must support integrated, ‘land sharing’ approaches that enhance fallow management. In more expansive upland areas, enhancing integrated fallow management would involve working with upland farmers to support variegated swidden fallows that are agroecologically productive in ways that support crops yields (for subsistence and market sales) and the co-produced ecosystem services. As such, broader land-sharing approaches that formally demarcate spaces wherein enhanced long fallow can coexist with other land uses would give government, NGOs and farmers the opportunity to realise the productive potential of long-fallow systems over time. Our evidence suggests that, rather than being seen as anathema to productivity and sustainability, LFS must be reframed as a legitimate source of livelihood, agroecological renewal, and sustainable upland agriculture (Dove 1983; Fox et al. 2000). As van der Ploeg (2014) notes, upland farmers must play a greater role in defining the trajectory of their agriculture with a higher degree of autonomy over land and food production relative to other more coercive approaches.

National policies and practices must consider where swidden can continue in broader landscape mosaics. Policy-based zoning schemes can provide the appropriate spaces and incentives that allow for more extensive land uses to flank but not be encroached upon by other spaces wherein farmers adopt perennial and annual cash cropping. In the right upland conditions, the prospect of sharing lands in this way makes it possible to maintain residual swidden forest cover in the varied ‘in-between’ spaces of medium-sized plantations and smallholder farms—a dynamic that has long existed as part of ‘composite’ swidden systems (Rambo 1998) in Southeast Asia. However, such spatial (re)arrangement will only succeed if it is legislated in spatially explicit policies—that is, policy advocating tenurial security in specific swidden fallow zones. The potential for long-fallow swidden to support agroecologically diverse landscapes is thus most feasible when supported by formal state policies that advocate land-sharing approaches.

While the prospect of promoting or creating space for long-fallow swidden remains challenging in the context of agrarian change, it has considerable potential to maintain and enhance livelihood security and ecosystem services. The results of this study indicate an urgent need to amend forest governance and agricultural policies that advocate the full incorporation and/or displacement of upland swidden peoples into intensified production schemes. Rather than maintain policies that seek to prohibit and often criminalise swidden, broader landscape approaches to forest governance would keep farmers on their land, working with fallowing systems that enhance rather than deplete ecosystem services and livelihood security. Such an approach stands the greatest chance of sustainable upland agriculture and climate change mitigation for the future.

## Electronic supplementary material

Below is the link to the electronic supplementary material.

Supplementary material 1 (PDF 252 kb)

## Footnotes

1 While we acknowledge that the livelihood security and ecological functions of LFS documented in earlier studies are less apparent today, the fact that these empirical details were documented and continued to be examined in situ (not necessarily as part of a transitional process) suggests indigenous, and smallholder communities do retain swidden fallow systems for the reasons described above (see Cairns 2007). Clearly, however, cases documenting the contribution of LFS to livelihood security and ecosystem services are becoming fewer.

2 Mendeley and Endnote.

3 To ensure that all reviewers applied the inclusion and exclusion criteria consistently, 500 titles were randomly selected to conduct an ‘inter-rater’ agreement Fleiss’ Kappa (k) Test (Fleiss, 1971). Three reviewers screened 200 articles at the title level in the first kappa test, yielding a k score of 0.54, below the required threshold of 0.6. After discussing discrepancies and application of the inclusion/exclusion criteria among reviewers, a second kappa test was run, resulting in a k score of 0.61, permitting progress to full title screening. A similar process, including two rounds of kappa testing, was done at abstract level screening, resulting in a kappa score of 0.6.

4 LFS is greater than or equal to 5 years fallow, while SFS is less than 5 years fallow.

5 fsQCA 2.5 software uses the Quine-McCluskey algorithm to arrive at a ‘solution’ or combination of drivers which are likely to produce a given outcome (Ragin 2008). In order to judge the performance of each analysis and whether results are meaningful and acceptable, two important indicators were used: coverage and consistency. Coverage indicates what percentage of cases (studies) are covered by the given solution and consistency indicates the level of consistency with which that solution explains the outcome (i.e. the significance). If consistency scores are <1 then some studies deviate from the general pattern identified and therefore scores over 0.7 are considered acceptable.

6
http://arohatgi.info/WebPlotDigitizer/app/?.

7
http://www.cebm.brown.edu/open_meta.

8 Forest plots are a widely recognised, standard way of illustrating graphically the results of a meta-analysis, see http://www.cebi.ox.ac.uk/for-practitioners/what-is-good-evidence/how-to-read-a-forest-plot.html.

9 Detailed descriptions of drivers and outcomes including sample and exemplary text from specific studies can be found in Table S5.

10 Figures used to present results are forest plots which summarise the effect sizes when comparing data from long-fallow swidden systems and alternative land uses. Each plot shows results for each study (including author, date, transition type, and unique ID reference) with full details of the study provided in Table S6. The forest plot shows an effect size estimate and overall summary effects. The boxes on the plot range in size depending on their weight, allocated based on sample size and the horizontal lines represent the 95 % confidence interval. The dotted line through the diamond shows the summary effect across all studies which is used in interpreting the entire dataset. Details of overall heterogeneity across study results and p-values are also included in parentheses.

11 For AGC, individual study results were presented in a variety of units for both biomass and carbon. These were converted to a standard AGC estimate and compared using a standardised mean difference (SMD) treatment based on 12 data points across 3 studies.

12 Results for the upper soil horizon were highly heterogeneous (and thus not presented here) but did show a small net increase in bulk density under alternative land uses. In particular, when the effects of transition to perennial cash crops (pepper, oil palm and rubber) were isolated, there was a statistically significant and moderate increase in bulk density with a level of heterogeneity just outside our defined threshold of 75 %.

13 This is corroborated to some extent by the QCA results which showed that for transitions from LFS to shorter fallow systems, in almost 80 % of cases where intensification was cited as a major driver, a decline in soil fertility was reported.

14 This variation by soil depth is not surprising as we would anticipate the effects of land use to be greater in the upper soil horizons whereas at greater depths, other factors such as underlying geology, leaching, and soil formation processes might be a greater influence than the effect of land management and crop type.

15 The exception is when all agriculture (including swidden) is removed completely to allow for full forest regeneration, but the net effect will depend on where the swidden farmers are relocate and what they do there.

16 The list of indicators is not exhaustive but rather suggestive of the types of information and data extracted from studies.
