# Supplementary material for: The impact of swidden decline on livelihoods and ecosystem services in Southeast Asia: A review of the evidence from 1990 to 2015
Source: Ambio. 2016 Nov 16;46(3):291–310. doi: 10.1007/s13280-016-0836-z (PMC5347523; doi:10.1007/s13280-016-0836-z)
Supplement: Supplementary file 1 — Supplementary material 1 (PDF 252 kb) [file 13280_2016_836_MOESM1_ESM.pdf]

Ambio: Electronic Supplementary Material (ESM)

This supplementary material has not been peer reviewed.

Title: The impact of swidden decline on livelihoods and ecosystem services in Southeast Asia: a review of the evidence from 1990 to 2015

Authors: Wolfram Dressler, David Wilson, Jessica Clendenning, Rob Cramb, Rodney Keenan, Sango Mahanty, Thilde Bech Bruun, Ole Mertz, Rodel D. Lasco

Contents

Table S1. Literature Search Results

Table S2 List of studies included in the final review

Table S3. Critical Appraisal Tool

Table S4 Risk of Bias Tool

Table S5 Summary of drivers and outcomes with explanations for each category

Table S6: Details of studies included in the quantitative meta-analysis.

Table S1. Literature Search Results

Ref	Database/Source	Date Search Conducted	# of results	Search terms, strings and combinations used	Search Period
1	Agricola	08-Jul-14		swidden OR shifting OR Rotation* OR "slash and burn" AND livelihood* OR cash OR production OR yield OR income AND ecosystem OR soil OR water OR carbon AND upland AND south east asia OR Philippines OR Thailand OR Malaysia OR Indonesia OR Cambodia OR Laos OR Papua New Guinea	n/a
2	ScienceDirect	08-Jul-14	207	(Swidden OR shifting OR Rotation* OR "slash and burn") AND (livelihood*) AND (ecosystem) AND (upland) AND (Thailand OR Vietnam OR “Viet Nam” OR Cambodia OR Laos OR Philippines OR Indonesia OR Myanmar OR Burma OR Malaysia OR “Papua New Guinea” OR “Southeast Asia”)	1945 - Present
3	Springlink	08-Jul-14	213	(Swidden OR shifting OR Rotation* OR "slash and burn") AND (livelihood*) AND (ecosystem) AND (upland) AND (Thailand OR Vietnam OR “Viet Nam” OR Cambodia OR Laos OR Philippines OR Indonesia OR Myanmar OR Burma OR Malaysia OR “Papua New Guinea” OR “Southeast Asia”)	1945 - Present
4	Directory of Open Access Journals (DOAJ)	09-Jul-14	6	swidden OR shifting OR Rotation OR "slash and burn" AND Asia	Any
5	Directory of Open Access Journals (DOAJ)	09-Jul-14	1	swidden OR shifting OR Rotation OR "slash and burn" AND Philippines	Any
6	Directory of Open Access Journals (DOAJ)	09-Jul-14	1	swidden OR shifting OR Rotation OR "slash and burn" AND Laos	Any
7	Directory of Open Access Journals	09-Jul-14	4	swidden OR shifting OR Rotation OR "slash and burn" AND Vietnam	Any

Ref	Database/Source	Date Search Conducted	# of results	Search terms, strings and combinations used	Search Period
	(DOAJ)				
8	AGRIS: International Information System of the Agricultural Science and Technology (FAO)	09-Jul-14	15	swidden OR shifting OR Rotation* OR "slash and burn" AND south east asia OR Philippines OR Thailand OR Malaysia OR Indonesia OR Cambodia OR Laos OR Papua New Guinea	Any
9	Web of Science (v.5.13.2)	10-Jul-14	4,579	Search Strings 1, 1a, 2 and 3	1945 - 2014
10	Web of Science (v.5.13.2)	10-Jul-14	3,867	Search Strings 1, 2 and 3a	1945 - 2014
11	Ebrary	10-Jul-14	129	swidden OR shifting OR Rotation* OR "slash and burn" AND livelihood* OR cash OR production OR yield OR income AND ecosystem OR soil OR water OR carbon AND upland AND south east asia OR Philippines OR Thailand OR Malaysia OR Indonesia OR Cambodia OR Laos OR Papua New Guinea	Any
12	US Forestry: TreeResearch	10-Jul-14	8	shifting cultivation	Any
13	FAO Catalogue Online	10-Jul-14	8	swidden	Any
14	British Library	10-Jul-14	35	shifting cultivation AND asia	Any
15	British Library	10-Jul-14	29	swidden AND asia	Any
16	British Library	10-Jul-14	17	slash and burn AND asia	Any
17	British Library	10-Jul-14	14	Forest fallow AND Asia	Any
18	British Library	11-Jul-14	47	Upland Agriculture AND Asia	Any
19	World Bank Open Knowledge Repository	11-Jul-14	8	"shifting cultivation" AND asia	Any
20	World Environment Library collection	11-Jul-14	7	"shifting cultivation"	Any
21	UN University Library Catalogue	11-Jul-14	5	"swidden OR "shifting cultivation" OR "slash and burn" AND "southeast asia"	Any

Ref	Database/Source	Date Search Conducted	# of results	Search terms, strings and combinations used	Search Period
22	Agricola (articles)	11-Jul-14	10	slash and burn	Any
23	Agricola (articles)	11-Jul-14	37	shifting cultivation	Any
24	Agricola (articles)	11-Jul-14	5	swidden	Any
25	Agricola (books)	11-Jul-14	20	slash and burn	Any
26	Agricola (books)	11-Jul-14	13	shifting cultivation	Any
27	Agricola (books)	11-Jul-14	103	swidden	Any
28	PlosOne	14-Jul-14	26	(((everything:swidden) OR everything:"slash and burn") OR everything:"shifting cultivation") AND everything:"southeast Asia"	Any
29	FAO Publications	14-Jul-14	12	swidden OR "Shifting Cultivation" OR "slash and burn" AND southeast Asia	Any
30	Wil de Jong (Response to Literature Call)	16-Jul-14	6	n/a	n/a
31	Judith Mayer (Response to Literature Call)	16-Jul-14	16	n/a	n/a
32	Carol Colfer (Response to Literature Call)	16-Jul-14	19	n/a	n/a
33	'Christel' (Response to Literature Call)	16-Jul-14	4	n/a	n/a
34	Google	17-Jul-14	72	swidden OR shifting OR Rotation* OR "slash and burn" AND upland AND southeast asia OR Philippines OR Thailand OR Malaysia OR Indonesia OR Cambodia OR Laos OR Papua New Guinea	Any

Ref	Database/Source	Date Search Conducted	# of results	Search terms, strings and combinations used	Search Period
35	CAB	15-Jul-14	548	1 and 2 and 3<br>(Thailand OR Vietnam OR “Viet Nam” OR Cambodia* OR Laos OR “Lao PDR” OR Philippines OR Myanmar OR Burma OR Malaysia* OR “Papua New Guinea” OR “Southeast Asia”) AND (Swidden* OR “Slash AND burn” OR “shifting cultivation” OR “forest fallow*” OR fallow* OR “upland farm*” OR rotation* OR farm* OR regrowth OR “hill tribe” OR tradition* OR integral OR mixed) AND (Livelihood* OR income OR crop OR production OR yield OR portfolio OR wealth OR surplus OR risk OR vulnerability OR inequality OR diversif* OR speciali$ation OR needs)	Any
36	CAB	15-Jul-14	357	1 and 2 and<br>3aThailand OR Vietnam OR “Viet OR Nam” OR cambodia* ORLaos OR “Lao OR P DR” OR Philippines OR Myanmar ORBurma OR malaysia* OR “Papua AND New OR Guinea” OR“Southeast AND Asia” AND swidden* AND “Slash ANDburn” OR “shifting OR cultivation” OR “forest ORfallow*” OR fallow* OR “upland OR farm* ” OR rotation* ORfarm* OR regrowth OR “hill OR tribe” OR tradition* ORintegral OR mixed AND ecosystem* OR "Environmentalservice" OR "natural resource*" OR soil OR carbon	Any
37	JSTOR	18-Jul	877	(((("Southeast Asia") AND (Swidden* OR "shifting cultivation" OR fallow)) AND (Livelihood* OR income)) AND (soil or carbon))	Any
38	Wiley Online Library	20-Jul-14	337	1 and 3 and 3a (full text) and 2 (abstract)	1945-2014

Ref	Database/Source	Date Search Conducted	# of results	Search terms, strings and combinations used	Search Period
39	Wiley Online Library			Thailand OR Vietnam OR “Viet Nam” OR Cambodia* OR Laos OR “Lao PDR” OR Philippines OR Myanmar OR Burma OR Malaysia* OR “Papua New Guinea” OR “Southeast Asia” in FullText AND Swidden* OR “Slash and burn” OR “shifting cultivation” OR “forest fallow*” OR fallow* OR “upland farm*” OR rotation* in Abstract AND Livelihood* OR income OR crop OR production OR yield OR portfolio OR wealth OR surplus OR risk OR vulnerability OR inequality OR diversif* OR speciali$ation in FullText AND ecosystem* OR "Environmental service" OR "natural resource*" OR soil OR carbon in FullText between years 1945 and 2014	
40	Scopus	20-Jul-14	7,138	1 and 1a and 2 and 3 and 3a	Any
41	Scopus			thailand OR vietnam OR "Viet Nam" OR cambodia* OR laos OR "Lao PDR" OR philippines OR myanmar OR burma OR malaysia* OR "Papua New Guinea" OR "Southeast Asia" OR "Greater Mekong" AND uplands OR montaneOR slop* OR elevate* OR mountain* OR hill* AND swidden* OR "Slash and burn" OR "shifting cultivation" OR "forest fallow*" OR fallow* OR "upland farm*" OR rotation* OR indigenous AND livelihood* OR income OR crop ORproduction OR yield OR portfolio OR wealth OR surplus OR risk OR vulnerability OR inequality OR diversif* AND ecosystem* OR "Environmental service" OR "natural resource*" OR soil OR carbon	Any
45	Google Scholar	22-Jul	1570	Normal search: "shifting cultivation" or "swidden" or "slash and burn" and "southeast Asia"	any

Ref	Database/Source	Date Search Conducted	# of results	Search terms, strings and combinations used	Search Period
46	National Agriculture and Forestry Research Institute (NAFRI): LAO Agriculture Database	22-Jul	10	Swidden	any
47	National Agriculture and Forestry Research Institute (NAFRI): LAO Agriculture Database	22-Jul	22	"Shifting cultivation"	Any
48	Malaysia Journal of Tropical Forest Science	22-Jul	2	Swidden	Any
49	Malaysia Journal of Tropical Forest Science	22-Jul	2	"Shifting cultivation"	Any

Ref	Database/Source	Date Search Conducted	# of results	Search terms, strings and combinations used	Search Period
50	ProQuest via the Swedish Uni for Agricultural Sciences	12-Aug	1839	loc(Thailand OR Vietnam OR "Viet Nam" OR Cambodia* OR Laos OR "Lao PDR" OR Philippines OR Myanmar OR Burma OR Malaysia* OR "Papua New Guinea" OR "Southeast Asia" OR "Greater Mekong" OR zamia OR Borneo OR "Indonesia* archipelago" OR "Indo-China" OR Malaya OR Kampuchea OR Sarawak OR "British North Borneo" OR "insular south east Asia" OR "Netherlands East Indies" OR Siam OR "peninsular Malaysia" OR Pacific) AND ab(swidden* OR "Slash and burn" OR "shifting cultivation" OR "forest fallow*" OR fallow* OR "upland farm*" OR rotation* OR farm* OR barbarian OR "ethnic minorit*" OR indigenous OR custom* OR pioneer* OR "forest eaters" OR montane OR imperator OR "enriched fallow" OR "long fallow" OR "forest-fallow" OR "rural poor" OR peasant OR "forest farm*" OR agriculture* OR regrowth OR "hill tribe" OR tradition* OR integral OR mixed) AND ab(Livelihood* OR income OR crop OR production OR yield OR portfolio OR wealth OR surplus OR risk OR vulnerability OR inequality OR diversify* OR speciali action OR needs OR capital OR education OR remit* OR migration OR relocaction OR dependency OR subsisted* OR cash OR market OR credit OR debt OR loan* OR land OR rich OR poor OR disparity OR secure* OR mortality OR nutrition OR deaths OR morbidity OR sufficient* OR asset* OR access OR food OR staple OR trade OR "non-timber forest product*" OR "non-wood forest product*" OR "minor forest product*" OR NTFP OR wildlife OR game OR bushmeat OR "bush meat" OR fish OR hunt* OR bird*) AND all(ecosystem* OR "Environmental service" OR "natural resource*" OR soil OR carbon)	Any

Table S2 List of studies included in the final review

UNID	DOC ID	Author(s)	Publication Year	Journal/Publication	Publication Title
001	6877	Ole Mertz & Kelvin Egay & Thilde Bech Bruun & Tina Svan Colding	2013	Human Ecology	The Last Swiddens of Sarawak, Malaysia
002	16574	T. S. Hansen & O. Mertz	2006	Land Degradation & Development	Extinction Or Adaptation? Three Decades Of Change In Shifting Cultivation In Sarawak, Malaysia
003	16574	T. S. Hansen & O. Mertz	2006	Land Degradation & Development	Extinction Or Adaptation? Three Decades Of Change In Shifting Cultivation In Sarawak, Malaysia
004	1086	Doolittle, Amity A.	2001	Human Ecology	From Village Land to “Native Reserve”: Changes in Property Rights in Sabah, Malaysia, 1950–1996
005	1915	Terauchi, D. et al.	2014	Open Journal of Forestry	Implication for Designing a REDD+ Program in a Frontier of Oil Palm Plantation Development: Evidence in East Kalimantan, Indonesia
006	4894	Evans, Phanvilay, K., Fox, J., Vogler, J.	2011	Journal of Land Use Science	An agent-based model of agricultural innovation, land-cover change and household inequality: the transition from swidden cultivation to rubber plantations in Laos PDR
007	5201	Belsky and Siebert, S.F	2003	Agriculture and Human Values	Cultivating cacao: Implications of sun-grown cacao on local food security and environmental sustainability
008	5229	Forsyth& Evans, N.	2012	World Development	What is Autonomous Adaption? Resource Scarcity and Smallholder Agency in Thailand
009	5349	Seidenberg, Mertz and Kias	2003	Geografisk Tidsskrift-Danish Journal of Geography	Fallow, labour and livelihood in shifting cultivation: Implications for deforestation in northern Lao PDR
010	6102	Feintrenie, L., S. Schwarze, and P. Levang.	2010	Ecology and Societ	Are local people conservationists? Analysis of transition dynamics from agroforests to monoculture plantations in Indonesia
011	7475	Ducourtieux, O	2006	NAFRI Workshop Proceedings	SHIFTING CULTIVATION AND POVERTY ERADICATION: A COMPLEX ISSUE
013	16375	Linquist, B et al.	2007	Mountain Research and Development	Montane paddy rice: Development and effects on food security and livelihood activities of highland Lao farmers
014	16504	Pandey, S and van Minh	1998	Agriculture, Ecosystems and Environment	A socio-economic analysis of rice production systems in the uplands of northern Vietnam

UNID	DOC ID	Author(s)	Publication Year	Journal/Publication	Publication Title
015	16558	Dressler, W and Pulhin, J	2010	Agriculture and Human Values	The shifting ground of swidden agriculture on Palawan Island, the Philippines
016	16589	Nyuk-Wo Lim, J. and Douglas, I.	1998	Asia Pacific Viewpoint	The impact of cash cropping on shifting cultivation in Sabah, Malaysia
017	16643	Vongvisouk, T. et al.	2014	Applied Geography	Shifting cultivation stability and change: Contrasting pathways of land use and livelihood change in Laos
018	16689	Cramb, R.A. et al.	2009	Human Ecology	Swidden Transformations and Rural Livelihoods in Southeast Asia
019	16689	Cramb, R.A. et al.	2009	Human Ecology	Swidden Transformations and Rural Livelihoods in Southeast Asia
020	16689	Cramb, R.A. et al.	2009	Human Ecology	Swidden Transformations and Rural Livelihoods in Southeast Asia
021	16899	Hariyadi, B and Ticktin, T.	2012	Human Ecology	From Shifting Cultivation to Cinnamon Agroforestry: Changing Agricultural Practices Among the Serampas in the Kerinci Seblat National Park, Indonesia
022	17207	Olivier Ducourtieux,et al.	2005	Development and Change	Land Policy and Farming Practices in Laos
023	17198	Mucahid Mustafa Bayrak, Tran Nam Tu and Paul Burgers	2015	Book Chapter (Cairns, 2015, eds.)	Formal and Indigenous Forest-Management Systems in Central Vietnam
025	7486	Yokoyama, S	2003	PhD Thesis	Geographical study on the basis for existence of mountainous Villages in northern Laos
026	7551	Rerkasem and Rerkasem	1994	IIED Report	Shifting Cultivation in Thailand : its current situation and dynamics in the context of highland development Faculty of Agriculture terms of what was most relevant to the particular country .
027	7551	Rerkasem and Rerkasem	1994	IIED Report	Shifting Cultivation in Thailand : its current situation and dynamics in the context of highland development Faculty of Agriculture terms of what was most relevant to the particular country .
028	7551	Rerkasem and Rerkasem	1994	IIED Report	Shifting Cultivation in Thailand : its current situation and dynamics in the context of highland development Faculty of Agriculture terms of what was most relevant to the particular country .
029	7811	Kanazawa, H., Hoshikawa, K. and Nawata, E.	2006	Japanese Journal of Tropical Agriculture	The changes in land use and agricultural systems of Karen people in North Thailand - a case study of Sam

UNID	DOC ID	Author(s)	Publication Year	Journal/Publication	Publication Title
					Sop Bon village
030	7461	Roberts, M	2011	PhD Thesis	Traditional Beliefs and Farmer Decision- Making in the Uplands of Northern Lao PDR
031	712	Grange & Kansuntisukmongkol	2004	SuperSoil Proceedings	Impact of fallow length on soil structure and soil water characteristics in a swidden cultivation system of western Thailand
032	4972	Tanaka Tachibe, S et al	2009	Agriculture, Ecosystems and Environment	Soil characteristics under cash crop farming in upland areas of Sarawak, Malaysia
033	7374	Neergaard, Magid and Mertz	2008	Agriculture, Ecosystems and Environment	Soil erosion from shifting cultivation and other smallholder land use in Sarawak, Malaysia
034	15004	Chaplot, V. , Le Bissonnais, Y. and Bernadou, J	2006	T&F Book Chapter	Runoff, soil, and soil organic carbon losses within a small sloping-land catchment of Laos under shifting cultivation
035	15579	Saito, K. et al.	2006	Plant Soil	Cropping intensity and rainfall effects on upland rice yields in northern Laos
036	16308	Woo et al.	2011	Agriculture, Ecosystems and Environment	Stand structure and natural regeneration of degraded forestland in the northern mountainous region of Vietnam
037	16308	Woo et al.	2011	Landscape Ecol Eng	Stand structure and natural regeneration of degraded forestland in the northern mountainous region of Vietnam
038	16148	Sillitoe, P	1996	Singapore Journal of Tropical Geography	FALLOW AND FERTILITY UNDER SUBSISTENCE CULTIVATION IN THE PAPUA NEW GUINEA HIGHLANDS: II. SOIL FERTILITY
039	16392	Funakawa, S. et al	1997	Soil Science and Plant Nutrition	Physicochemical properties of the soils associated with shifting cultivation in northern Thailand with special reference to factors determining soil fertility
040	16392	Funakawa, S. et al	1997	Soil Science and Plant Nutrition	Physicochemical properties of the soils associated with shifting cultivation in northern Thailand with special reference to factors determining soil fertility
041	16392	Funakawa, S. et al	1997	Soil Science and Plant Nutrition	Physicochemical properties of the soils associated with shifting cultivation in northern Thailand with special reference to factors determining soil fertility
042	16548	Aumtong, S. et al	2009	Agriculture, Ecosystems and Environment	Relating soil carbon fractions to land use in sloping uplands in northern Thailand
043	16551	Dupin, B et al.	2009	Soil & Tillage Research	Assessment of tillage erosion rates on steep slopes in northern Laos

UNID	DOC ID	Author(s)	Publication Year	Journal/Publication	Publication Title
044	16744	Bruun, T.B. et al.	2013	Agriculture, Ecosystems and Environment	Improved sampling methods document decline in soil organic carbon stocks and concentrations of permanganate oxidizable carbon after transition from swidden to oil palm cultivation
045	16691	Watanabe, E. et al.	2004	Southeast Asian Studies	Soil fertility and farming systems in a slash and burn cultivation area of Northern Laos
046	17211	Tanaka, S et al.	1997	Soil Science and Plant Nutrition	Soil ecological study on dynamics of K, Mg, and Ca, andsoil acidity in shifting cultivation in Northern Thailand
047	7482	Kenzo, T et al	2010	Forest Ecology and Management	Changes in above-and belowground biomass in early successional tropical secondary forests after shifting cultivation in Sarawak, Malaysia
048	16273	Silva Carreiras, J.M.B., Rosa, I., Pereira, J.M.C	2011	J. Geophys. Res	Greenhouse gas emissions from shifting cultivation in the tropics, including uncertainty and sensitivity analysis
049	17186	Bruun, T.B. et al.	2014	IREDD Project Report	Impacts of reducing emissions from deforestation and forest degradation and enhancement of forest carbon stocks
050	17186	Bruun, T.B. et al.	2014	IREDD Project Report	Impacts of reducing emissions from deforestation and forest degradation and enhancement of forest carbon stocks
051	16041	Van Dung, N et al.	2008	Agriculture, Ecosystems and Environment	Analysis of the sustainability within the composite swidden agroecosystem in northern Vietnam
052	17212	Chan et al.	2013	Forest Ecology and Management	Establishment of allometric models and estimation of biomass recovery of swidden cultivation fallows in mixed deciduous forests of the Bago Mountains, Myanmar
054	16629	Valentin, C. et al	2008	Agriculture, Ecosystems and Environment	Runoff and sediment losses from 27 upland catchments in Southeast Asia: Impact of rapid land use changes and conservation practices
055	16677	Lestrelin, G. et al.	2012	Natural Resources Forum	Challenging established narratives on soil erosion and shifting cultivation in Laos
056	4556	Wezel, A	2002	Land Degradation & Development	Temporal changes of resource use, soil fertility and economic situation in upland northwest Vietnam
057	15920	Tyynela, T. , Otsamo, R. and	2003	Agroforestry Systems	Indigenous livelihood systems in industrial tree-

UNID	DOC ID	Author(s)	Publication Year	Journal/Publication	Publication Title
		Otsamo, A.			plantation areas in West Kalimantan, Indonesia: Economics and plant-species richness
058	1861	Nguyen Patanothai, A., Rambo, A.T.	2004	Southeast Asian Studies	Recent changes in the composite swidden farming system of a Da Bac Tay ethnic minority community in Vietnam's northern mountain region
059	4262	McCarthy, J.F.	2010	Journal of Peasant Studies	Processes of inclusion and adverse incorporation: Oil palm and agrarian change in Sumatra, Indonesia
061	17189	Lawrence, D. et al.	2007	Book Chapter (Cairns, 2007 eds.)	Does Tree Diversity Affect Soil Fertility? Findings from Fallow Systems in West Kalimantan
062	9173	La-Orngoplew, W	2012	PhD Thesis	Living under the rubber 'boom' : market integration and agrarian transformations in the Lao uplands.
063	11516	Martin Rudbeck Jepsen	2006	Forest Ecology and Management	Above-ground carbon stocks in tropical fallows, Sarawak, Malaysia
064	16785	Bruun, T.B. et al.	2006	Agriculture, Ecosystems and Environment	Linking yields of upland rice in shifting cultivation to fallow length and soil properties
065	16544	Roder et al.	1995	Plant and Soil	Relationships between soil, fallow period, weeds and rice yield in slash-and-burn systems of Laos
066	17216	Funakawa, S. et al	1997	Soil Science and Plant Nutrition	Ecological study on the dynamics of soil organic matter and its related properties in shifting cultivation systems of Northern Thailand
067	17216	Funakawa, S. et al	1997	Soil Science and Plant Nutrition	Ecological study on the dynamics of soil organic matter and its related properties in shifting cultivation systems of Northern Thailand
068	17216	Funakawa, S. et al	1997	Soil Science and Plant Nutrition	Ecological study on the dynamics of soil organic matter and its related properties in shifting cultivation systems of Northern Thailand
069	17215	Catherine Hepp	2013	Copenhagen Thesis	The Ecological Sustainability of Short Fallow Shifting Cultivation in Upland Systems
101	1105	Wolfram Dressler & Melanie McDermott & Will Smith & Juan Pulhin	2012	Human Ecology	REDD Policy Impacts on Indigenous Property Rights Regimes on Palawan Island, the Philippines
102	1940	Mucahid Mustafa Bayrak, Tran Nam Tu,	2013	Journal of Political Ecology	Restructuring space in the name of development: the sociocultural impact of the Forest Land Allocation

UNID	DOC ID	Author(s)	Publication Year	Journal/Publication	Publication Title
		and Paul Burgers			Program on the indigenous Co Tu people in Central Vietnam
103	8082	William D. Sunderlin,<br>Ida Aju Pradnja Resosudarmo,<br>Edy Rianto, and<br>Arild Angelsen	2000	CIFOR Occasional Paper	The Effect of IndonesiaÕs Economic Crisis<br>on Small Farmers and Natural Forest Cover<br>in the Outer Islands
104	7890	Dendi, A. et al	2004	Land Degradation & Development	Evolution of the Minangkabau's shifting cultivation in the West Sumatra highland of Indonesia and its strategic implications for dynamic farming systems
105	8002	Palao, L K M, et al	2011	Asia Life Sciences	Changes in indigenous land use system: from swidden to sedentary agriculture
106	8072	Penot , E	2004	Book Chapter	From shifting agriculture to sustainable complex rubber agroforestry systems (jungle rubber) in Indonesia: a history of innovation processes
107	8129	de Jong, W	2001	Book Chapter: Agricultural Technologies and Tropical Deforestation	The impact of rubber on the forest landscape in Borneo
109	8936	Alberny, E	2009	Masters Thesis	Dynamics of rubber plantation in Lao PDR
110	14766	Cohen, P.T.	2000	The Geographical Journal	Resettlement, opium and labour dependence: Akha-Tai relations in northern Laos
111	16497	Wadley, R.L. and Mertz, O.	2005	Agricultural Systems	Pepper in a time of crisis: Smallholder buffering strategies in Sarawak, Malaysia and West Kalimantan, Indonesia
112	17001	Castella, J.C. et al	2006	Land Use Policy	Impact of forestland allocation on land use in a mountainous province of Vietnam
113	17184	Koczberski, G. , Curry, G.N. and Bue, V	2012	Asia Pacific Viewpoint	Oil palm , food security and adaptation among smallholder households in Papua New Guinea
114	16640	Hansen, T.S.	2005	Singapore Journal of Tropical Geography	Spatio-temporal aspects of land use and land cover changes in the Niah catchment, Sarawak, Malaysia
115	16675	Folving, R. and Christensen, H.	2007	Geografisk Tidsskrift-Danish Journal of Geography	Farming system changes in the Vietnamese uplands - Using fallow length and farmers' adoption of Sloping Agricultural Land Technologies as indicators of environmental sustainability

UNID	DOC ID	Author(s)	Publication Year	Journal/Publication	Publication Title
118	17182	Castella, J.C. et al	2012	Human Ecology	Effects of Landscape Segregation on Livelihood Vulnerability: Moving From Extensive Shifting Cultivation to Rotational Agriculture and Natural Forests in Northern Laos
119	16617	D. D. Poudel, T. M. Nissen and D. J. Midmore	1999	Mountain Research and Development	Sustainability of commercial vegetable production under fallow systems in the uplands of Mindanao, the Philippines
121	8897	Colchester, M and Chao S	2011	Book	Oil Palm Expansion in South East Asia Trends and implications for local communities and indigenous peoples
201	1273	Ongprasert, Somchai & Prinz, Klaus	2004	Southeast Asian Studies	Intensification of shifting cultivation by the use of Viny legumes in Northern Thailand
202	3526	Jakobsen, Jens	2006	Geografisk Tidsskrift, Danish Journal of Geography	The role of NTFPs in a shifting culitivation systems in transition: A village case study from the uplands of North Central Vietnam
203	8957	Viau, Julien; Keophosay, Anousith; Castella, Jean Christophe	2011	Report	Impact of maize expansion on traditional rice production system in Northern Lao PDR
206	15611	Dove, Michael	1993	Economic Botany	Smallholder Rubber and Swidden Agriculture in Borneo: A sustainable adaptation to the ecology and economy of the tropical forest
207	15612	Turner, Sarah	2012	The Professional Geographer	Forever Hmong: Ethnic minority livelihoods and Agrarian transition in upland northern Vietnam
208	15718	Manivong, V.; Cramb, R.A.	2008	Book Chapter: D.J. Snelder and R.D. Lasco (eds.), Smallholder Tree Growing for Rural Development and Environmental Services.	The adoption of smallholder rubber production by shifting cultivators in northern Laos: A village case study
209	15850	Ducourtieux, Olivier	2006	Report	Is the diversity of shifting cultivation held in high enough esteem in Lao PDR?
210	16070	Bouahom, B; Douangsavanh, L; Rigg, J.	2004	Geoforum	Building sustainable livelihoods in Laos: untangling farm from non-farm, progress from distress
212	16118	Josol, R. C.; Montefrio, J.F.	2013	Agroecology and	Understanding the resilience of swidden agroecosystems

UNID	DOC ID	Author(s)	Publication Year	Journal/Publication	Publication Title
				Sustainable Food Systems	interacting with rubber and oil palm production regimes in the Philippines
213	16210	Padoch, C; Harwell, E, Susanto, A	1998	Human Ecology	Swidden, Sawah, and In-between: Agricultural transformation in Borneo
215	8938	Fitriana, Y. R.	2008	Masters Thesis	Landscape and farming system in transition: case study in Viengkham District, Luang Prabang Province, Lao PDR

Table S3. Critical Appraisal Tool

Quality Domain	Description/considerations	Score<br>[0=no, 1 = unclear; 2= yes]	Support for assessment<br>[*Copy text from study* and justify score]	Page & Paragraph
**Study Directness**	1. Does the study consider the population, exposure and comparators of interest in the review?	0		
	Sub total (max 2)	0	NOTE: If this Scores 0 then do not proceed any further as the article is excluded based on relevance	
**Conceptual Framing**	2. Does the study set out a theoretical or conceptual framework and hypothesis, theory or central argument which is tested?			
	Sub total (max 2)	0		
**Transparency**	3. Are details of the study location and population provided?			
	4. Are the aims and objectives of the research clearly stated?			
	Sub total (max 4)	0		

Quality Domain	Description/considerations	Score [0=no, 1 = unclear; 2= yes]	Support for assessment [*Copy text from study* and justify score]	Page & Paragraph
**Appropriateness**	5. Is the research design clearly specified and appropriate for the aims and objectives of the research?			
	6. Do the researchers display sufficient data to support their interpretations and conclusions?			
	7. Is the duration of study adequate?			
	Sub total (max 6)	0		
**Validity**	8. Measurement validity: Are selected indicators or measures appropriate for the stated outcome of interest given the local context?			
	9. Internal validity (Credibility): Are confounding variables or sources of bias identified and mitigated for?			
	10. External validity (Transferability): are the results or findings transferable to other contexts,			

Quality Domain	Description/considerations	Score [0=no, 1 = unclear; 2= yes]	Support for assessment [*Copy text from study* and justify score]	Page & Paragraph
	locations and settings outside those in the study area?			
	Sub total (max 6)	0		
**Reliability**	11. Stability: has the study demonstrated appropriate application of stated measurement and consistency of data gathering?			
	Sub total (max 2)	0		
**Cogency**	12. Is the study consistent from proposing a conceptual framework through methodology, data gathering, results and conclusion?			
	Sub total (max 2)	0		
	*Total score (max 24)**	0		

***Quality Rating** 19-24: High Quality; 13>18: Moderate Quality; 7>12: Low Quality; 0-6: Very Low Quality

Table S4 Risk of Bias Tool

Bias Domain	Risk Rating	Support for assessment where available<br><br>[*Copy text* from study and include reasons for score]
SELECTION BIAS DUE TO INADEQUATE RANDOMISATION		
PERFORMANCE BIAS		
DETECTION BIAS		
ATTRITION BIAS DUE TO INCOMPLETE OUTCOME DATA		
REPORTING BIAS DUE TO SELECTIVE REPORTING		
OTHER BIAS		
SUMMARY ASSESSMENT:		

Table S5 Summary of drivers and outcomes with explanations for each category

Driver/Outcome	Aggregated Category (QCA Code)	Constituent Category	Description	Sample Text (Study ID)
**Underlying Driver**	Land Use Policies (LUP)	Land Use Policies	Policies which determine the use of and governance over land including land allocation i.e. Forest and Land Use Planning or Land Use Planning laws in Vietnam and Laos	*The conversion from shifting cultivation areas to rubber plantations is driven by the government policy that aims to reduce shifting cultivation (UNID 17)*
**Underlying Driver**	State/NGO Agricultural Intervention (AG_Ext)	Extension	Government/non-government led schemes or activities designed to support, incentivise or even enforce a transition away from swidden agriculture through provision of technical guidance.	*We found that the economic incentives evolving from the changing market environments along with the improved access to the modern agricultural inputs, namely fertilizers and improved varieties of the wetland rice, as well as access to the agricultural extension services were major driving forces of the rapid expansion of wetland rice cultivation.(UNID 104)*
		Technology	Technological interventions such as irrigation infrastructure and improved germplasm which are designed to support a transition away from Swidden.	*The technology transfer in the village is primarily villager-to-villager rather than through an agricultural extension agent, although Chinese entrepreneurs have been an emerging source of technical information. (UNID 111)*
		Subsidy/Incentive	Government provided cash or agricultural materials (seeds, equipment etc) to adopt a new crop or practice	*local government officials also provided small amounts of pesticide free-of-charge to farmers who cultivated rai (UNID 8)*
**Underlying Driver**	Population Increase (POP)	Population Increase	Observed increase in absolute population or population density in the area of interest. T	*population growth, resettlement and land reform have had a very significant impact on farmers' access to land in Ban Lak Sip, engendering a ten-fold increase in population density per unit of agricultural land over the last quarter of a century and propelling a rapid intensification of the shifting cultivation system.(UNID 55)*
**Underlying Driver**	Market Influence (MAR_INF)	Market Influence	Market based drivers which could include access to markets via intermediaries, increased prices for products and incentive schemes from government/other actors to increase production of	*Moreover, increased trade and market integration with neighboring countries (mainly China, Vietnam, and Thailand) and the partial commoditization of land has spurred many local people to shift from subsistence*

Driver/Outcome	Aggregated Category (QCA Code)	Constituent Category	Description	Sample Text (Study ID)
			a certain crop.	*based to market-based production.*
**Underlying Driver**	Expanding Infrastructure (INFRA)	Expanding Infrastructure	This usually refers to roads, especially those linking communities to markets	*The improvement and construction of rural roads and relocation of many villages to these roads in the 1970s further helped to tie shifting cultivators more permanently to the market economy. (UNID 16)*
**Underlying Driver**	Commodity Price Fluctuation (CPF)	Commodity Price Increase	Increase in commodity process interpreted as a signal to increase production of a given crop. Also refers to commodity price shocks which force producers to abandon a crop. Signals a change towards intensification of cash crops because of better prices/linked to markets	*the rising crop prices and demand made the Tagbanua farmers further venture into fruit cropping, as they usually plant fruit crops after planting upland rice in their swidden plots.(UNID 105)*
		Commodity Price Decrease	Decrease in commodity price (often sudden i.e. a shock) which drives a change in practice and or crop selection/arrangement. A cause of insecurity and perhaps better captured in the outcome “single crop dependency”	*Farmers were dismayed by the low amount they received and the level of debt associated with these entitlements. Most decided to sell during the economic crisis when they faced a collapse in rubber prices (UNID 59)*
		New Crop selection in response to Market Demand	Alternative land use i.e. new crop choice driven by increased market demand i.e. inflated price of rubber/pepper etc.	*It turned out that market adjustment of monetary value of the Indonesian Rupiah (Rp.) against the US dollar substantially increased the farm-gate price of exportable crops such as rubber, patchouli, cocoa, gambir, etc., therefore creating an incentive for the cultivation of these crops (UNID 104)*
**Proximate Condition**	Individualisation of tenure (IND_TEN)	Individualisation of tenure	Customary land rights give way to private ownership of land (by either an individual or family) which drives transitions away from traditional uses towards activities which increase individual household income and benefits.	*The new forest classifications have also brought new types of management and control. Previously, land use had always been organized through the village patriarch. Now, local people must apply for rights to long-term use of forest land at commune level, through government agencies like Commune People’s Committees and Forest Management Boards.*
**Proximate Condition**	Intensification (INTFY)	Intensification: land policy	Land use (either swidden or the alternative land use) becomes more intensified as a result of a policy which restricts, alters or influences the type or extent of swidden practice.	*With market integration, agricultural and livestock products have provided important income sources for some households that have decreased their reliance on forest products and expanded and intensified the area*

Driver/Outcome	Aggregated Category (QCA Code)	Constituent Category	Description	Sample Text (Study ID)
				used for agriculture to the detriment of the forest. Old fallows and secondary forests have been turned into permanent crops.(UNID 118)
		Intensification (other/not specified)	This category was used when there had been an intensification of some kind but that could not be directly linked to an underlying policy. This was often the case in quantitative ecosystem services paper which describe an intensification but no driver.	*Thus overall, this increased cropping intensity with 2 years of cultivation followed by 2 years of fallow and associated with the replacement of upland rice by Job's tear and maize, led to an increase in mean annual total sediment yield (5.9 Mg ha 1 year 1) or a 600% increase when compared to the previous system*
**Proximate Condition**	Restriction of LFS livelihood activities (RESTRICT_LH)	Restriction of LFS livelihood activities	E.g. The article says the ban on opium and timber felling caused farmers to go to the mountain to clear more areas for rice paddies and corn. The point is that it was a major cash source.	*"I remember one time many police came to this village to check every family.They stated, 'Stop growing opium, if you do not stop we will take your buffaloes, your pigs, your horses, all of them will be taken. We will go up to your room [rice storage], and we will take it all.' " Not surprisingly, Lue noted that this had caused considerable resentment among local Hmong residents, as their revenues declined rapidly. (UNID 207)*
**Proximate Condition**	Reduction in Upland Rice Productivity (RED_UPRICE)	Reduction in Upland Rice Productivity	Productivity of existing swidden system (of which upland rice is an integral part) is reduced for an unspecified reason but it is this which precipitates the outcome/s identified.	*According to respondents, since rubber was introduced to Hadyao, upland rice cultivation had changed significantly. Nearly 75 percent reported that they cultivated a smaller area of upland rice after planting rubber. (UNID 208)*
**Proximate Condition**	Restricted Land Access (RESTRICT_LAND)	Restricted Land Access	Physical restriction to land via enforced allocation, enclosure, tenure arrangements etc.	*among the 29 farmers who reported decreasing yields, 23 mentioned short fallow periods as the main proximate cause, while soil erosion was cited only once. (UNID 55)*
**Outcome**	Reduced access to fallow land (RED_ACC_FALLOW)	Reduced access to fallow land	Fallow land availability and access decreased	*LUPLA also aimed at substituting individual land certificates for customary land tenure arrangements (i.e., right of clearance or 'axe rights'). Each household was allocated three upland plots, which meant that the maximum fallow period possible was limited to two years.(UNID 118)*

Driver/Outcome	Aggregated Category (QCA Code)	Constituent Category	Description	Sample Text (Study ID)
**Outcome**	Land Conflict (LAND_CONFL)	Land Conflict	Changes in swidden practice or adoption of alternative land use leads to conflicts over access to and rights over land within and between commnities and external actors.	*"Our respondents explain that aside from immediate economic needs, the growth of oil palm has influenced the way they make decisions about their land. First, indigenous swidden farmers sell their lands to prospective oil palm growers (mostly outsiders) in order to evade conflict with neighboring landowners." (UNID 212)*
**Outcome**	Reduction of customary practices (RED_CUSTOM)	Reduction of customary practices	This could be the change in roles of the village headman or rights, rituals and practices associated with swidden	*This [shift from swidden] has resulted in a loss of traditional knowledge as well as traditional forest management practices, all cornerstones in traditional Co Tu society.(UNID 102)*
	Reduced Socio-Cultural Wellbeing (RED_SOC)	Social problems increased	Increased social issues documented e.g. alcohol, drugs, crime, etc)	*In LongSegar and its offshoot communities, social problems have increased (drinking, drugs, prostitution, theft). There is more conflict (over ethnicity, rights, resources), and there is less security of access to resources. Their territory has been steadily squeezed by logging, plantations, and transmigration (UNID 18)*
		Constraints on local decision making	Autonomy to make own decisions about how land is managed and crop selection is reduced because of alternative land use	*The second non-financial concern was the difficulty of keeping autonomy of the villagers. Their autonomy would be threatened with respect to the selection of shipments as well as constraints regarding the change of land use choice, interculture and rigid work regulations. (UNID 5)*
		Increased or re-enforced Inequality	Existing inequality due to financial or social differences is exacerbated or new inequality emerges	*the inclusion of cash crops and livestock and the development of the paddy area have led to household inequality and stratification whereby some have access to more luxuries, i.e. power, television, motorcycles. (UNID 69)*
		Health or nutrition worsened	Worsening health of communities involved in the transition	*Although many Akha who settled on the lower slopes have improved their economic prospects through access to wet-rice land Ð especially the villages of Ban*

Driver/Outcome	Aggregated Category (QCA Code)	Constituent Category	Description	Sample Text (Study ID)
				Sopi Mai and Ban Yang Luang discussed above Đ these gains have been largely negated by increased human and livestock mortality that has accompanied resettlement.(UNID 110)
**Outcome**	Increase in income from alt. LU (INCOME_UP)	Increase in income from alt. LU	> Income	*About 69 percent of the households mentioned that their highest ranking income source was rubber. Hence rubber had clearly become the major source of income in the village.(UNID 208)*
		Increase in remittance	> Income from external sources	*About 28 percent of households reported that their only source of cash income was selling tub-lump rubber; 29 percent only earned income from other sources (livestock, other cash crops, selling rubber seedlings, working for wages, and from relatives in the USA); the remaining 43 percent received income from both rubber and other sources.(UNID 208)*
**Outcome**	Increased income diversification (INCOME_DIV)	Increased income diversification	Increase in number and type of income sources. This is not valued i.e. not considered positive or negative	*Diversification is back in favour as systems become more mixed, but it is back from a strictly economic, rather than an ecological or traditional, perspective.*
**Outcome**	Narrowing of livelihood options (NARROW_LH)	Narrowing of livelihood options	Forced to sell livestock, NTFPS, illegal logs etc for cash/rice as a result of swidden transition.	*They are, it could be said, facing an existential squeeze: declining availability of food is causing villagers to turn to the market to meet their needs; but a concomitant decline in cash generation from NTFPs and livestock is also compromising their ability to meet this shortfall through market exchange.(UNID 62)*
		Less diversified Livelihood strategies	i.e. shifting more towards monocropping or reliance on single livelihood component	*Smallholders, for example, have possibly traded long term food security for short-term income gains by expanding the area of oil palm production, thereby leaving themselves more vulnerable to the vagaries of world market prices for palm oil (UNID 113)*
**Outcome**	Single crop dependency (MONO_CROP)	Increase in vulnerability: single crop dependency	Reliance on one crop and the removal of the buffer provided by swidden agriculture	*Farmers dependent on monocultured oil palm plantations can become vulnerable to socioeconomic shocks, as such system does not permit the same level of response diversity.(UNID 212)*

Driver/Outcome	Aggregated Category (QCA Code)	Constituent Category	Description	Sample Text (Study ID)
**Outcome**	Decline in staple self-sufficiency/yield (DEC_STAPLE)	Decline in staple self-sufficiency/yield	Staple component of swidden system is reducing	*The decreasing fallow periods and restrictions in access to forest areas lay another reason for the decreasing swidden productivity (UNID 105).*
**Outcome**	Decreased food security (DEC_FOODSEC)	Decreased food security	This category is only for those cases in which food security. In our analysis we will need to define what we mean by food security or a number of categories are combined under food security i.e staples, diversification, labour productivity & nutrition etc?	*Food security will be negatively influenced by the decline in shifting cultivation as Ban Navene faces mandated restrictions without the provision of alternative livelihood options (UNID 69)*
**Outcome**	De-prioritising of upland rice or swidden crops (DEPRIOR_UPRICE)	De-prioritising of upland rice or swidden crops	Hill rice becomes supplementary (in place of rubber, paddy etc.)	*The landscapes mosaics have homogenized as maize replaced upland rice shifting cultivation system at the bottom of the hill sides and then progressed upwards towards the hill tops.(UNID 203)*
**Outcome**	Decline in soil fertility due to shorter fallow/cash crop (DEC_SOILF)	Decline in soil fertility due to swidden intensification	Soil parameters indicate a reduction in fertility owing to intensification of swidden system i.e. shortening of fallow.	*As the length of fallow period was shortened and soil fertility declined, the contribution of swiddening to household income decreased in later years.(UNID 58)*
		Decline in soil fertility due to cash crop intensification	Soil parameters indicate a **reduction** in fertility owing to intensification of cash crops replacing and when compared with swidden	*Intensification of land use per surface unit, as found in cash crops and perennial plantations, has also caused problems for maintaining soil fertility and the risk of soil erosion.(UNID 215)*
		Increased Sediment Yield	Sediment yield increases as a result of swidden transition. This is interpreted as a reduction in soil fertility as the top soil is lost	*Thus overall, this increased cropping intensity with 2 years of cultivation followed by 2 years of fallow and associated with the replacement of upland rice by Job's tear and maize, led to an increase in mean annual total sediment yield (5.9 Mg ha 1 year 1) or a 600% increase when compared to the previous system (UNID 54)*
**Outcome**	Decrease in forest	Decrease in forest cover/carbon	Decrease in forest cover and/or above ground carbon	*This statement was confirmed by our analysis of a chronological series of land-use maps (1954, 1977,*

Driver/Outcome	Aggregated Category (QCA Code)	Constituent Category	Description	Sample Text (Study ID)
	cover/carbon (DEC_FOR)			*1998) that revealed the rapid decrease in forest cover between 1977 and 1998 in the study area (UNID 112)*
**Outcome**	Increased Labor Input (INC_LAB)	Increased Labor Input	Labour demand is increased as a result of swidden transition – perhaps associated with additional labour required for weeds or labour being diverted to other non-staple related activities	*Informants considered fields made after short fallow to require more labour, which conflicts with records of labour input. The reason could be that clearing long fallow plots requires more or at least similar labour inputs compared to the labour needed for extra weeding after short fallow, and therefore overall labour requirements may in some cases be higher after long fallow. (UNID 9)*
**Outcome**	Decrease in labor productivity (DEC_LABPROD)	Decrease in labor productivity	Productivity of labour i.e. the amount of person hours/day/hectare required to produce the same amount of output (e.g. yield) has reduced	*This land use intensification entails a higher rate of increase in the agricultural labour input than the agricultural output, thereby causing a decline in labour productivity and food security (UNID 202)*
**Outcome**	Specialisation/redistribution of labour: on and Off Farm (LAB_SPEC)	Specialisation of labour	Labour is more specialised or redistributed to other tasks not associated with swidden	*During pepper price surges in the late 1980s and late 1990s, men stayed home to cultivate pepper; conversely, when prices dropped, men returned to long-distance wage work. Also evident in these data is a lag in men stopping wage work as prices rise, such that absences do not reach their lowest points until pepper prices have peaked.(UIND 111)*
**Outcome**	Increased biophysical constraints (BIO_CONSTR)	Increased Inputs: pesticide/fertilizer	Inputs required to maintain/enhance soil productivity or reduce pests/weeds have increased.	*Households who did not apply fertilizers mentioned that they were eager to apply them but did not have enough money. All the households wanted to apply more fertilizers if possible. More than 70 % of the households used herbicides. Most of them told that after fertilizer application, the increase in the weed population and resulting difficulty in controlling weeds had motivated them to start using herbicides (UNID 29)*
		Increase in weed pressure	Incidence of weeds or labour required to remove them has increased	*Local farmers consider that the fallow period is short if it is less than 6 years. They believe that short-fallow fields have more weeds. They have to weed four times*

Driver/Outcome	Aggregated Category (QCA Code)	Constituent Category	Description	Sample Text (Study ID)
				per crop incase of short-fallow, whereas only two weedings are needed in the case of long-fallow.(UNID 45)
**Outcome**	Criminalisation of swidden (CRIM_SWID)	Criminalisation of swidden	Swidden becomes criminalised as a result of polices and a transition away from traditional practice.	*In particular, the anti-swidden ordinances have had a lasting negative impact on the local subsistence security and agro-ecological diversity of swiddens. As a result of the ban, Tagbanua farmers who had cleared, but not yet burned and planted, their swiddens were left without harvests.(UNID 15)*

Table S6: Details of studies included in the quantitative meta-analysis. The 'UNID' can be used to cross reference with results presented in figures 4 – 6 in the main manuscript with full references included in Table S2 (this document).

UNID	DOC ID	Author	Year	Country	Transition Type	Specific Transition	Soil Type
32	4972	Tanaka Tachibe, S et al	2009	MY	LFS to Perennial Cash Crop	LFS to Oil Palm	Typic Dysrudepts
32	4972	Tanaka Tachibe, S et al	2009	MY	LFS to Perennial Cash Crop	LFS to Rubber	Typic Dysrudepts
32	4972	Tanaka Tachibe, S et al	2009	MY	LFS to Perennial Cash Crop	LFS to Pepper	Typic Dysrudepts
33	7374	Neergaard, Magid and Mertz	2008	MY	LFS to Perennial Cash Crop	LFS (Upland Rice) to Pepper	Tropudults
36	16308	Woo et al.	2011	VN	LFS to Forest	> 10 Year fallow to Plantation	n.d.
37	16308	Woo et al.	2011	VN	LFS to Forest	> 10 Year fallow to Plantation	n.d.
38	16148	Sillitoe, P	1996	PNG	LFS to Annual Cash Crop	>10 Year Fallow to > 5 Years Cash Crop Cultivation	Inceptisols
38	16148	Sillitoe, P	1996	PNG	LFS to Annual Cash Crop	>10 Year Fallow to > 10 Years Cash Crop Cultivation	Inceptisols
44	16744	Bruun, T.B. et al.	2013	MY	LFS to Perennial Cash Crop	LFS ->3yr Oil Palm (2002 vs 2011) 1a	Ultisols (FAO)
44	16744	Bruun, T.B. et al.	2013	MY	LFS to Perennial Cash Crop	LFS ->8yr Oil Palm (2002 vs 2011) 1a	Ultisols (FAO)
44	16744	Bruun, T.B. et al.	2013	MY	LFS to Perennial Cash Crop	LFS ->8yr Oil Palm (2002 vs 2011) 1b	Ultisols (FAO)
44	16744	Bruun, T.B. et al.	2013	MY	LFS to Perennial Cash Crop	LFS ->3yr Oil Palm (2002 vs 2011) 2a	Ultisols (FAO)
47	7482	Kenzo, T et al	2010	MY	LFS to Shorter Fallow	17 Year Fallow to 4 Year Fallow	Typic Kandihumult
49	17186	Bruun, T.B. et al.	2014	LA	LFS to Shorter Fallow	20 - 25 Year fallow to 7-10 year fallow	Ultisols
49	17186	Bruun, T.B. et al.	2014	LA	LFS to Shorter Fallow	20 - 25 Year fallow to Cultivated land	Ultisols
49	17186	Bruun, T.B. et al.	2014	LA	LFS to Shorter Fallow	20 – 25 Year fallow to 0 Year Fallow	Ultisols
49	17186	Bruun, T.B. et al.	2014	LA	LFS to Shorter Fallow	20 - 25 Year fallow to 3 - 4 year fallow	Ultisols
49	17186	Bruun, T.B. et al.	2014	LA	LFS to Shorter Fallow	7 - 10 Year fallow to 0 Year Fallow	Ultisols
50	17186	Bruun, T.B. et al.	2014	IN	LFS to Annual Cash Crop	6 Year Fallow to Continuous Cultivation	Ultisols
50	17186	Bruun, T.B. et al.	2014	IN	LFS to Perennial Cash Crop	6 Year Fallow to rubber	Ultisols
52	17212	Chan et al.	2013	MN	LFS to Shorter Fallow	30 Year Fallow to 5 Year Fallow	Ultisols
52	17212	Chan et al.	2013	MN	LFS to Shorter Fallow	25 Year Fallow to 5 Year Fallow	Ultisols
52	17212	Chan et al.	2013	MN	LFS to Shorter Fallow	20 Year Fallow to 5 Year Fallow	Ultisols
52	17212	Chan et al.	2013	MN	LFS to Shorter Fallow	30 Year Fallow to 1 Year Fallow	Ultisols
52	17212	Chan et al.	2013	MN	LFS to Shorter Fallow	25 Year Fallow to 1 Year Fallow	Ultisols
52	17212	Chan et al.	2013	MN	LFS to Shorter Fallow	20 Year Fallow to 1 Year Fallow	Ultisols
61	17189	Lawrence, D. et al.	2007	IN	LFS to Perennial Cash Crop	19 Year Fallow to Agroforestry	Clay Rich

UNID	DOC ID	Author	Year	Country	Transition Type	Specific Transition	Soil Type
							Ultisols/Oxisols
64	16785	Bruun, T.B. et al.	2006	MY	LFS to Shorter Fallow	25 Year Fallow to 0 Year Fallow: Amat	Tropudults
64	16785	Bruun, T.B. et al.	2006	MY	LFS to Shorter Fallow	14 Year Fallow to 0 Year Fallow: Amat	Tropudults
69	17215	Catherine Hepp	2013	LA	LFS to Shorter Fallow	11 Year Fallow to 3 Year fallow	Ultisol
69	17215	Catherine Hepp	2013	LA	LFS to Shorter Fallow	11 Year Fallow to 5 Year fallow	Ultisol
69	17215	Catherine Hepp	2013	LA	LFS to Shorter Fallow	11 Year Fallow to 2 Year fallow	Ultisol
39-41	16392	Funakawa, S. et al	1997	TH	LFS to Shorter Fallow	> 8 Year Fallow to < 4 Year Fallow	Ustic Dystropepts
39-41	16392	Funakawa, S. et al	1997	TH	LFS to Annual Cash Crop	LFS to vegetable cultivation (0 Fallow)	Ustic Dystropepts
